# Trisk 95 as a novel skin mirror for normal and diabetic systemic glucose level

**DOI:** 10.1038/s41598-020-68972-6

**Published:** 2020-07-22

**Authors:** Nsrein Ali, Hamid Reza Rezvani, Diana Motei, Sufyan Suleman, Walid Mahfouf, Isabelle Marty, Veli-Pekka Ronkainen, Seppo J. Vainio

**Affiliations:** 1 Laboratory of Developmental Biology, Faculty of Biochemistry and Molecular Medicine, Biocenter Oulu, University of Oulu, Aapistie 5A, 90220 Oulu, Finland; 2 Inserm, BMGIC, UMR 1035, University of Bordeaux, Bordeaux, France; 3 Centre de Référence pour les Maladies Rares de la Peau, CHU de Bordeaux, Bordeaux, France; 4 Inserm U1216, Grenoble Institut des Neurosciences, University Grenoble, La Tronche, France; 5 Biocenter Oulu, University of Oulu, 90220 Oulu, Finland; 6 Infotech Oulu, University of Oulu, 90220 Oulu, Finland; 7 Borealis Biobank of Northern Finland, Oulu University Hospital, Oulu, Finland

**Keywords:** Cell biology, Cell signalling

## Abstract

Developing trustworthy, cost effective, minimally or non-invasive glucose sensing strategies is of great need for diabetic patients. In this study, we used an experimental type I diabetic mouse model to examine whether the skin would provide novel means for identifying biomarkers associated with blood glucose level. We first showed that skin glucose levels are rapidly influenced by blood glucose concentrations. We then conducted a proteomic screen of murine skin using an experimental in vivo model of type I diabetes and wild-type controls. Among the proteins that increased expression in response to high blood glucose, Trisk 95 expression was significantly induced independently of insulin signalling. A luciferase reporter assay demonstrated that the induction of Trisk 95 expression occurs at a transcriptional level and is associated with a marked elevation in the Fluo-4AM signal, suggesting a role for intracellular calcium changes in the signalling cascade. Strikingly, these changes lead concurrently to fragmentation of the mitochondria. Moreover, Trisk 95 knockout abolishes both the calcium flux and the mitochondrial phenotype changes indicating dependency of glucose flux in the skin on Trisk 95 function. The data demonstrate that the skin reacts robustly to systemic blood changes, and that Trisk 95 is a promising biomarker for a glucose monitoring assembly.

## Introduction

In view of its dynamic behaviour in the presence of multiples stress, the human skin is widely used to test cellular and molecular responses to specific treatments. Mirroring the biological and physiological internal changes in healthy and disease environments via the skin is the new revolutionary approach. The paradigm is based on the skin ultrastructure, consisting a sensory neuron network in tight connection with the skin keratinocytes^1^. Moreover, there are numerous clinical studies describing the skin features related to pancreas diseases^2,3^, rheumatic disorders^4^ and lung diseases^5^. Recent reports have revealed the presence of specific biomarkers in the skin that may serve for early diagnosis of neuronal conditions such as Alzheimer’s and Parkinson’s diseases^6^. In addition, acute or chronic ulcers, which are the most widely described skin features related to type I diabetes, are associated with reduced skin wound healing and an increased risk of infections^7–10^. These facts have meant that new biomarkers and non-invasive devices for glucose monitoring in the skin have emerged on the market in the last decade^11–14^. In fact, the currently most widely used devices require skin penetration as far as the interstitial fluid, and are thereby invasive, although a new generation of devices based on the measurement of various metabolites in sweat such as sodium, lactate, potassium and glucose has been proposed recently^15–18^. One major drawback with such devices, however, is the amount of sweat required, resulting in secondary effects such as skin irritation. In view of all the devices and the skin features to high glucose, we propose a new paradigm that operates by converting the skin keratinocytes cells to serve as biosensors for monitoring blood glucose.

Keratinocytes are the main cell type (≈ 90%) constituting the epidermis^19,20^, and with respect to their key roles in wound healing^21^, they have been extensively studied, notably in cases of diabetes^22–24^. Reports have demonstrated that impaired keratinocytes function can result in delayed wound healing, and that multiple physiological processes in keratinocytes, such as proliferation^25^, migration^26^, apoptosis^27^ and differentiation^28^, may be affected by a hyperglycaemic condition. Moreover, the skin barrier dysfunction^29,30^ and increased inflammation^31–33^ brought about by a hyperglycaemic environment will prevent the keratinocytes from healing; probably leading to continuously infected severe skin lesions^34,35^. In view of the literature, skin keratinocytes could be used as indicators of the status of normal and diabetic skin.

We set out here to investigate whether blood glucose concentration could be monitored by means of keratinocytes and whether this sensing activates specific biomarkers and their signalling pathways. Our results show that Trisk 95 expression is increased in the skin and its primary keratinocytes following an increase in blood glucose concentration. In turn, upregulation of Trisk 95 is associated with an increase in the intracellular calcium level, which then triggers a particular modification in the morphology of the mitochondrial network.

Our data suggest that keratinocytes could serve as promising biological sensors for developing a new generation of blood glucose monitoring devices, especially for use with diabetic patients.

## Results

### Skin glucose levels are rapidly influenced by glucose concentrations in the blood

**Figure 1:**
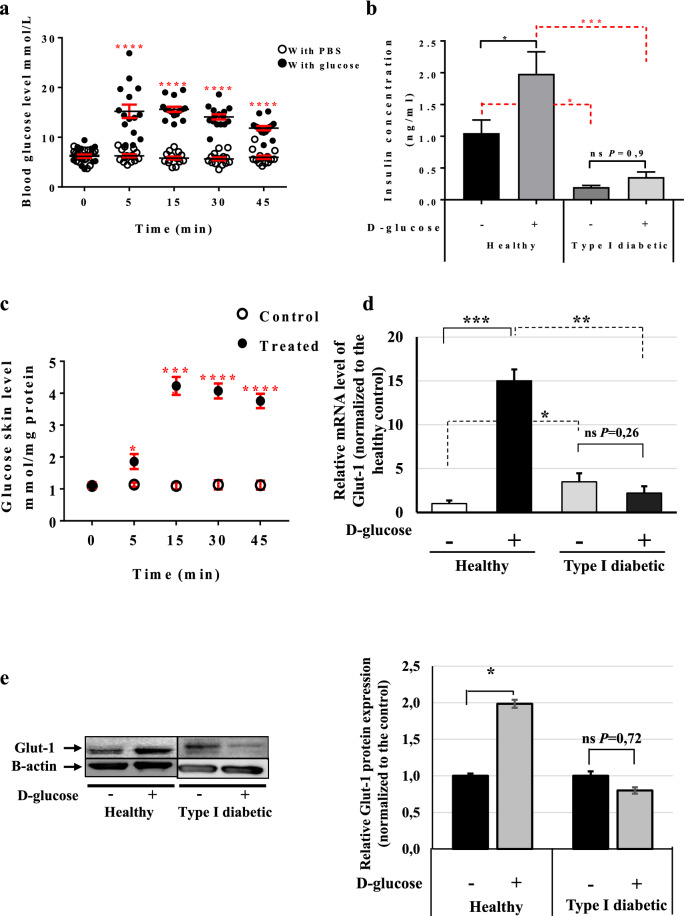
Skin rapidly senses any increase in blood glucose. (**a**) GTT assay using C57BL/6 mice. The mice were divided into two groups: the control group (empty circle) was injected IP with PBS, and the treated group (full circle) with 2 g/kg d-glucose diluted in PBS. Blood glucose levels were monitored at the given time points (n = 15 mice/group). (**b**) Insulin concentrations in healthy and type I diabetic mice. Insulin levels at 45 min post-injection were measured using the Insulin Rodent Chemiluminescence ELISA technique (n = 6 mice/group). (**c**) Skin glucose levels in vivo. The GTT assay was performed and skin biopsies were collected at 0, 5, 15, 30 and 45 min post-injection (n = 7 mice/group). Glucose levels were measured using the YSI 2,950 Biochemistry Analyzer (YSI Life Sciences) and normalized to the total concentration of proteins (mmol/mg protein). (**d**) *Glut-1* mRNA levels at 45 min post-injection in the skin of both healthy and type I diabetic mice was quantified by qRT-PCR. The results are shown as averages after normalization to the controls ± SD (n = 6/group). (**e**) Glut-1 protein expression level at 45 min post-injection was determined then quantified by western blotting and b-actin was used as a loading control. Two-way ANOVA analysis was performed in a using GraphPad Prism software, ****P < 0.0001 and (**c**) (*P < 0.02; ****P < 0.0001 respectively). One-way ANOVA was used for multiple comparisons in (**b**) (*P < 0.03; *P < 0.041; ***P < 0.0002, *ns* not significant 0.9, respectively). Two-tailed Student’s test was used in (**d**) (*P < 0.01; **P < 0.0094; ***P < 0.0003, respectively), and (**e**) (*P < 0.023, *ns* not significant 0.72)*.*

Little is known about the responses of epidermal cells to increased blood glucose concentrations or whether a particular signalling network is activated in these cells. To test this hypothesis, we first injected glucose into healthy and type I diabetic mice, the latter having been prepared as previously described^36^. Briefly, C57BL/6 mice were divided into two groups: (healthy) controls, which were injected intraperitoneally with citrate buffer (pH 4.5), and type I diabetic mice, which received one single dose of 150 mg/kg streptozotocin (STZ). Blood glucose was monitored every 48 h and the mice were considered diabetic when its level was ≥ 15 mmol/l (data not shown). The first step was to select a suitable end-point at which the blood glucose level in the healthy mice reaches that found in the diabetic mice (≥ 15 mmol/l), as this can be used for collecting the skin samples. For this purpose, we performed a glucose tolerance test (GTT) on the healthy mice. After fasting for 12 h, the mice were divided into two groups: a control group in which PBS was injected intraperitoneally, and a treatment group that received 2 g/kg d-glucose. Serum was collected from both groups (15 animals/group) at time-points 0, 5, 15, 30 and 45 min and blood glucose levels were monitored. The results showed a significant increase in blood glucose at 5 min post-injection in the glucose-injected mice (15.20 mmol/l ± 1.36) (Fig. 1a, full circle) as compared with the placebo-treated mice (6.27 mmol/l ± 0.31) (Fig. 1a, empty circles). The glucose concentration remained at this level up to the 45 min time-point and then started to drop (Fig. 1a). However, no changes in the blood glucose were observed in the glucose- and placebo-injected type I diabetic mice (Supplementary Fig. 1a and Table 1).

**Table 1:** Blood glucose level in type I diabetic mouse model. GTT assay was performed by using two groups: the control group was injected with PBS, and the treated group with 2 g/kg d-glucose.

Blood glucose level (mmol/L)/Time in min					
Mouse number	0	5	15	30	45
PBS injcetion					
1	27.3	26.2	33	33	30
2	27.4	27.3	33	33	33
3	28.4	33	33	33	33
4	29.6	32.6	33	32.9	33
5	29	31.6	32.4	33	33
6	28.3	30.6	33	33	33
7	33	33	33	33	33
8	30.2	32	33	33	33
d-glucose injection					
1	28.8	32.7	33	33	33
2	28.1	33	33	33	33
3	29.4	26.3	33	33	33
4	30.4	27.4	33	33	33
5	17	28.7	27.6	33	30.9
6	33	33	33	33	33
7	26.5	33	33	33	33
8	22.6	33	32.1	33	33
9	33	33	37.9	33	33
10	33	33	33	33	33

Blood glucose levels were monitored at the given time points (n = 8 mice for control and n = 10 for treated).

Measurement of the blood insulin concentrations at 45 min after glucose injection showed a significant increase in its level in the glucose-injected healthy mice (1.98 ng/ml ± 0.34) versus (1.05 ng/ml ± 0.20) in the placebo-injected mice, whereas the blood insulin concentration in the type I diabetic mice did not differ between those receiving a placebo or a glucose injection (0.35 ng/ml ± 0.08; and 0.20 ng/ml ± 0.026, respectively) (Fig. 1b).

To determine the impact of the increase of blood glucose concentrations on that of the skin, the skin glucose levels were monitored at the different time-points following glucose injection. Results showed that the skin glucose levels were increased gradually with a peak at 15 min after glucose injection in healthy mice (Fig. 1c). The skin glucose concentration remained at this level up to 45 min after glucose injection, as observed for the blood glucose level. However, no changes in the skin glucose level were observed between placebo-injected type I diabetic mice (Supplementary Fig. 1b). Comparing the skin glucose concentrations among four groups revealed that a significantly much higher amounts of glucose detected in the skin of the placebo- and glucose-injected type I diabetic mice compared to the healthy mice at different time-points (Supplementary Fig. 1c,d). To find the mechanism by which blood glucose is taken up by the skin, we examined the expression of glucose transporter-1 (Glut-1) at the mRNA and protein levels at 45 min post-injection. Our data demonstrated a 15-fold increase in *Glut-1* mRNA in the healthy mice after the glucose injection (Fig. 1d), while the Glut-1 protein level was upregulated (Fig. 1e).

Examination of Glut-1 expression in the diabetic mice indicated that glucose injection affected neither its mRNA expression nor its protein expression (Fig. 1d,e and Supplementary Fig. 1e,f). On the other hand, the *Glut-1* mRNA expression level in the placebo-injected type I diabetic mice was 3.4-fold higher than in the placebo-injected healthy ones (Fig. 1d). This may explain why the glucose level is significantly higher in the skin of diabetic mice than in that of healthy mice. Taken together, these results suggest that a modification in blood glucose concentration will regulate the skin glucose level in the same direction.

### Trisk 95 is a novel biomarker for detecting glucose in the skin

We then examined whether a specific pathway is activated in the skin upon an increase in blood glucose concentration. In respect to our finding showing that both blood and skin glucose levels are starting to decrease at 45 min, as well as the fact that 45 min is an optimal period for the glucose diffusion in the skin^37–39^.

**Figure 2:**
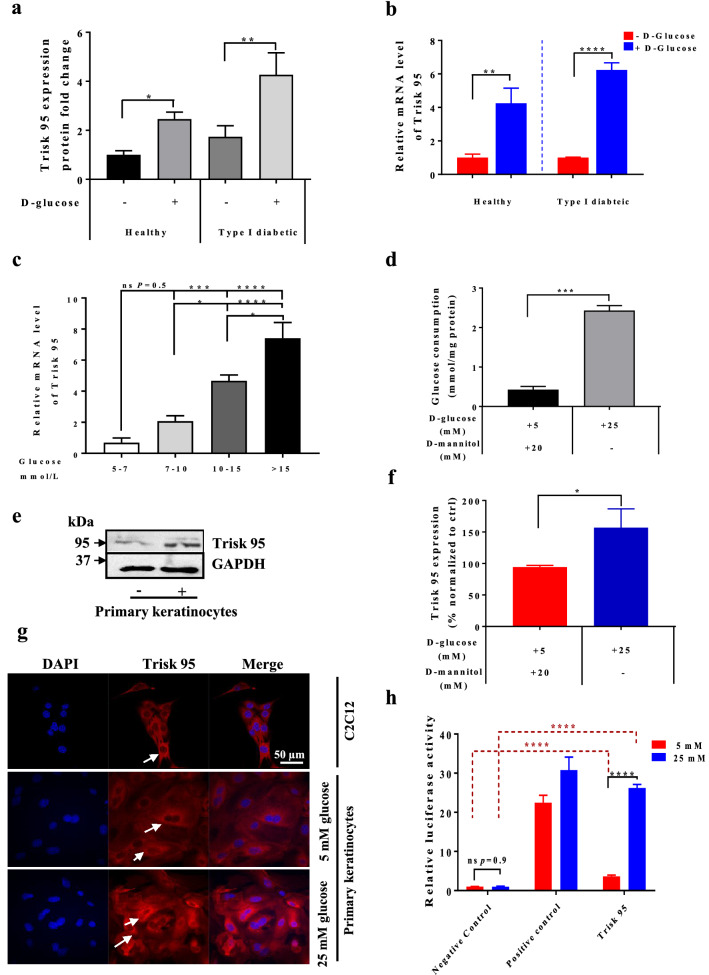
Trisk 95 is a new skin sensor for high blood glucose concentrations. (**a**) Proteomic profile of the skin after glucose injection in vivo. Skin biopsies collected at 45 min post-injection from four groups were subjected to proteomic analysis. The results are presented as ratios of the fold change after normalization to the healthy mice injected with PBS (n = 5/group). (**b**) *Trisk 95* mRNA expression level were quantified in the skin at 45 min after glucose injection using qRT-PCR. The results are shown as averages normalized to the control ± SD (n = 6 in the healthy group and n = 5 in the type I diabetic group). (**c**) *Trisk 95* mRNA expression levels were quantified in the healthy mouse skin at 45 min after injection of different glucose concentrations. The results are shown as averages normalized to the control ± SEM (n = 4, 6, 7 and 5, respectively). (**d**) Glucose consumption in vitro using primary keratinocytes. Glucose consumption was measured and normalized to protein concentrations. Data are presented as means ± SEM from three separate experiments. (**e**) Western blot analysis of Trisk 95 in primary keratinocytes. (**f**) Quantification of Trisk 95 protein expression levels in the primary keratinocytes. The results are shown as averages after normalization to the control ± SEM in three separate experiments. (**g**) Detection of Trisk 95 (red) and DAPI (blue) by immunofluorescence. The C2C12 cell line was used as a positive control. Trisk 95 expression was observed in the ER of myoblasts as well as in the keratinocytes (white arrows), the signal being stronger in the cells incubated with high glucose than in the controls. Scale bar = 50 µm. (**h**) Triadin as a promotor of high glucose. Relative luciferase activities are presented as means ± SEM after normalization to the negative controls (n = 3 in separate experiments conducted in triplicate). Two tailed Student’s t-test was used in (**a**) (*P < 0.02; **P < 0.002), (**b**) (**P < 0.005; ****P < 0.0001), (**d**) (***P < 0.0002) and (**f**) (*P < 0.02). One-way ANOVA multiple comparison was used in (**c**) (*P < 0.01; ***P < 0.0008; ****P < 0.0001, *ns* not significant *P* = 0.53). Two-way ANOVA multiple comparison was used in (**h**) (*ns* not significant. 0.99, ****P < 0.0001).

We, therefore, performed label-free differential proteomic analyses of the skin biopsies taken from the placebo and glucose-injected healthy and type I diabetic mice at 45 min post-injection. The results highlight the proteins that were significantly increased in three groups of comparisons (G2 vs G1, G4 vs G1, and G4 vs G3) (Supplementary Fig. 2a and Table 2). Based on our approach consisting on the identification of the potential biomarkers that respond as glucose-dependent and insulin-independent signalling, we found that Triadin isoform-1 (Trisk 95) was the protein among all others that significantly increased in both the healthy and type I diabetic groups after glucose injection (Fig. 2a). To investigate further the effect of blood glucose concentration on Triadin expression, *Trisk 95* mRNA levels were measured in the skin of the different groups of mice at 45 min post-injection.

**Table 2:** The effect of the glucose injection on the proteomic profiles in both of the healthy and diabetic mice.

Accession	Protein name	Peptides used for quantification	Confidence score	Fold change					T.Test				
				G2/G1	G3/G1	G4/G1	G4/G2	G4/G3	G2/G1	G3/G1	G4/G1	G4/G2	G4/G3
E9Q9K5	Triadin	2.00	7.21	2.46	1.74	4.27	1.74	2.45	0.00	0.18	0.02	0.12	0.05
Q9WUP7	Ubiquitin carboxyl-terminal hydrolase isozyme L5	2.00	3.81	1.19	2.34	5.23	4.41	2.24	0.65	0.01	0.01	0.01	0.03
B2RQC6	CAD protein	3.00	9.95	1.67	4.39	7.59	4.55	1.73	0.18	0.00	0.00	0.00	0.02
O08808	Protein diaphanous homolog 1	3.00	6.81	1.48	13.21	22.60	15.28	1.71	0.52	0.00	0.00	0.00	0.02
Q8R1B4	Eukaryotic translation initiation factor 3 subunit C	3.00	11.09	1.41	4.96	7.54	5.34	1.52	0.44	0.00	0.00	0.00	0.04
Q9D024	Coiled-coil domain-containing protein 47	2.00	7.52	1.06	1.45	2.14	2.01	1.48	0.70	0.02	0.01	0.01	0.05
Q9CX56	26S proteasome non-ATPase regulatory subunit 8	4.00	10.95	1.20	1.57	2.30	1.91	1.46	0.34	0.02	0.00	0.00	0.00
P0CW02	Lymphocyte antigen 6C1	2.00	6.16	1.01	1.61	2.25	2.21	1.39	0.92	0.02	0.00	0.00	0.04
Q9CQD1	Ras-related protein Rab-5A	7.00	19.41	0.99	1.35	1.86	1.87	1.38	0.95	0.00	0.00	0.00	0.02
Q99LD4	COP9 signalosome complex subunit 1	3.00	9.83	1.03	1.40	1.93	1.87	1.37	0.85	0.04	0.00	0.00	0.03
Q9R0Q9	Mannose-P-dolichol utilization defect 1 protein	2.00	5.09	1.11	1.83	2.32	2.09	1.27	0.62	0.01	0.00	0.00	0.02
Q3TPJ8	Cytoplasmic dynein 1 intermediate chain 2	6.00	19.15	1.05	1.24	1.56	1.49	1.26	0.68	0.02	0.00	0.01	0.03
O08529	Calpain-2 catalytic subunit	17.00	55.28	1.09	1.62	2.00	1.84	1.23	0.25	0.00	0.00	0.00	0.03
Q8BML9	Glutaminyl-tRNA synthetase	7.00	21.08	1.07	1.34	1.63	1.52	1.22	0.63	0.05	0.00	0.00	0.04
P14685	26S proteasome non-ATPase regulatory subunit 3	11.00	30.71	1.09	1.35	1.57	1.45	1.16	0.40	0.01	0.00	0.00	0.01
Q02053	Ubiquitin-like modifier-activating enzyme 1	45.00	158.02	1.00	1.19	1.37	1.37	1.15	0.97	0.02	0.00	0.00	0.04
Q8QZY1	Eukaryotic translation initiation factor 3 subunit L	11.00	35.46	1.31	2.03	2.30	1.76	1.13	0.14	0.00	0.00	0.00	0.02
Q921F2	TAR DNA-binding protein 43	5.00	18.21	1.18	1.88	1.92	1.62	1.02	0.04	0.00	0.00	0.00	0.78
Q99MR6	Serrate RNA effector molecule homolog	3.00	8.09	1.20	1.47	1.60	1.34	1.09	0.03	0.00	0.00	0.00	0.16
Q9DBR7	Protein phosphatase 1 regulatory subunit 12A	3.00	10.26	1.23	1.45	1.46	1.19	1.01	0.01	0.00	0.00	0.00	0.88
Q9JII5	DAZ-associated protein 1	4.00	12.29	1.35	1.52	1.73	1.28	1.14	0.04	0.00	0.00	0.03	0.10
Q91WK2	Eukaryotic translation initiation factor 3 subunit H	3.00	9.38	1.38	1.82	2.95	2.14	1.62	0.10	0.02	0.00	0.00	0.02

Description (Accession, protein name, peptides used for quantification, confident score, and fold change) of a set of proteins that are significantly increased in response to high glucose in the different comparisons (G2/G1, G3/G1, G4/G2, G4/G3). G2 and G4 represent the mice from healthy and type I diabetic that were injected with d-glucose (respectively), while G1 and G3 represent the mice rom healthy and type I diabetic that received PBS injection. Two-tailed Student’s t-test was used for statistical analysis and with *P* value of < 0.05 was considered as significant.

Consistent with the proteomic data, the results showed 3.45–4-fold increases in *Trisk 95* mRNA in both the healthy and type I diabetic mice receiving an intraperitoneal injection of glucose (Fig. 2b). Since the blood insulin concentration in type I diabetic mice does not change after a glucose injection, we concluded that the upregulation of Trisk 95 in the skin mediated by an increase in blood glucose takes place via an insulin-independent pathway. To further confirm this observation, we have injected the healthy mice using different glucose concentrations; 45 min post-injection *Trisk 95* mRNA expression levels were examined. Our finding demonstrated significant increases of *Trisk 95* mRNA expression that were associated with the increases of blood glucose in dependent manner (Fig. 2c). This data show a strong evidence that Trisk 95 expression is actively dependent of glucose-pathway.

Because the skin contains different cell types, and to further confirm specifically the Trisk 95 expression in primary mouse keratinocytes, we then cultured them in the presence of low (5 mM) and high (25 mM) d-glucose concentrations. In this experiment, primary keratinocytes isolated from healthy mice were cultured in a low-glucose medium for 10 days. The medium was then replaced with a fresh medium containing either a low (5 mM) or a high (25 mM) glucose level. Forty-five minutes later cell and medium samples were collected for further analysis. Measurement of the glucose uptake indicated a significant increase in glucose consumption when the cells had been incubated for 45 min in the presence of a high glucose concentration (2.43 ± 0.1 mmol/ug protein versus 0.43 ± 0.07 mmol/ug protein) (Fig. 2d), while *Trisk 95* mRNA and protein expression levels had likewise increased 2.3 and 1.6-fold, respectively (Supplementary Fig. 2b,c; Fig. 2e,f). Consistently with this, immunostaining of keratinocytes pointed to a significant increase in the expression of Trisk 95 (red) when cells were cultured in the presence of 25 mM d-glucose (Fig. 2g).

To further examine whether the glucose concentration affected the transcriptional expression of Trisk, we used a luciferase reporter plasmid in which the 882 bp region upstream of the ATG translation initiation codon of human *TRDN* had been cloned upstream of the luciferase. The results showed a significant increase in luciferase expression upon treatment of the cells with 25 mM d-glucose (Fig. 2h), indicating that the extracellular glucose concentration affects Triadin expression at the transcriptional level.

### An increased blood glucose concentration will trigger intracellular calcium level modifications

Given that Trisk 95 is a well-known actor in calcium release from the endoplasmic reticulum (ER) in skeletal and heart muscles^40–43^, we then wondered whether the Trisk 95 overexpression mediated by a high glucose concentration might affect the calcium level in the ER store. To answer this question, the basal level of intracellular calcium was imaged live using Fluo-4 AM 45 min after culturing primary mouse keratinocytes with either a low or a high-glucose medium.

**Figure 3:**
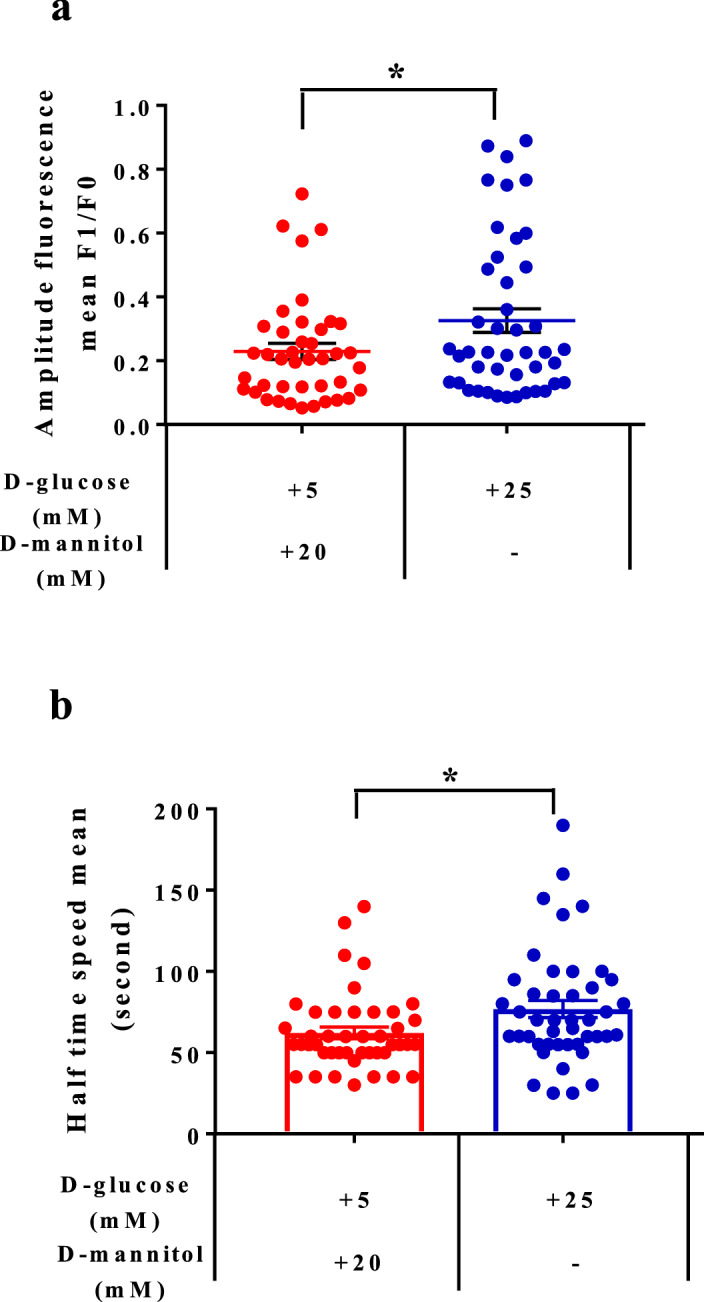
High extracellular glucose concentrations induce changes in the intracellular calcium in primary keratinocytes. (**a**) Intracellular calcium levels in primary keratinocytes after 45 min of glucose treatment. Cells were incubated for 30 min in the presence of Fluo-4 AM and intracellular calcium basal levels were measured for 50 s, after which thapsigargin was added to the medium. Time lapse recording was used to follow the increase in the fluorescence signal until the maximum amplitude was reached. The data showed a significant increase in the amount of cytosolic calcium in the cells incubated with high glucose relative to the controls (n = 41). (**b**) Speed required to reach the half maximum of the fluorescence signal. The keratinocytes incubated with high glucose were slower to reach their peak than were the controls. The two-tailed Student’s t-test was used for statistical analysis in (**a**) (*P < 0.01) and (**b**) (*P < 0.02).

To evaluate the release of calcium from ER, thapsigargin (an inhibitor of sacro/endoplasmic reticulum Ca2+ ATPase (SERCA) pump function) was added to block the pumping of calcium back into the ER. A significant increase in the maximum amplitude (background-subtracted F1/F0, where F0 is the minimum level of fluorescence before adding thapsigargin) was observed in the cells incubated with 25 mM d-glucose compared with the controls (0.32 ± 0.037 versus 0.2 ± 0.02) (Fig. 3a). We then measured the speed with which half of the maximum fluorescence amplitude was reached, and the results showed a significant difference between these two conditions, the speed being significantly slower in the cells incubated with high glucose (76.82 ± 5.28 s) than in the controls (62.16 ± 3.59 s) (Fig. 3b), suggesting that the release of calcium from the ER was higher under these conditions. Two representative movies showing the time lapse measurements with analysis of the regions of interest and the averaged fluorescence intensity traces under both sets of conditions are presented as supplementary data (Supplementary Fig. 3a,b).

### Primary keratinocytes exhibit alteration in mitochondrial network morphology due to the Trisk 95 upregulation

**Figure 4:**
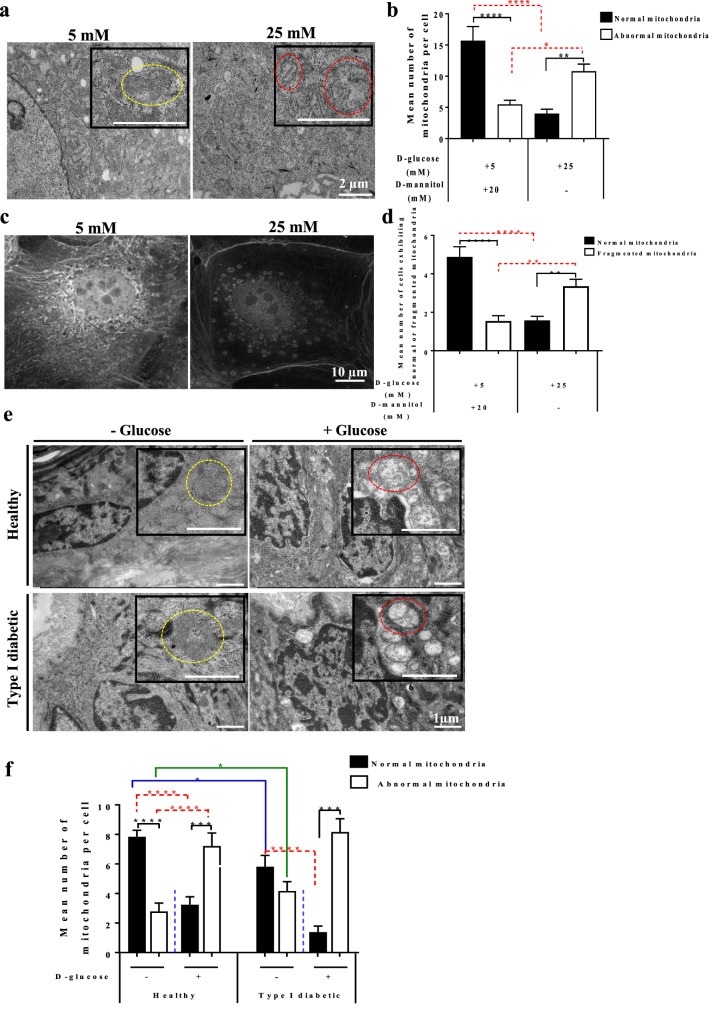
Keratinocytes exhibit a specific mitochondrial phenotype after high glucose treatment. (**a**) High extracellular glucose leads to an abnormal mitochondrial phenotype in keratinocytes. After 45 min of incubation in the presence of a low or a high-glucose medium, cells were fixed, and sections were examined using a Tecnai Spirit G2 transmission electron microscope. Keratinocytes incubated with high glucose demonstrated changes in the mitochondrial phenotype (swollen and enormous, round in shape, disarrangement of the cristae accompanied by partially or totally electron-lucent matrix) (red dotted circles); the normal mitochondria of different shapes (elongated or round) with well-arranged cristae and electron dense were observed in the cells incubated with low glucose (yellow dotted circles). Scale bar = 2 µm. (**b**) Quantification of the mitochondria confirms the histological observations. A significant increase in the abnormal mitochondria was noted in the cells incubated with high glucose (n = 45) compared with the controls (n = 51). (**c**) The mitochondrial network in the keratinocytes was visualized using MitoTracker staining. A neat mitochondrial network surrounding the nucleus was observed in the control cells, but a fragmented network presented itself in the cells treated with high glucose. (**d**) Quantification of normal and fragmented mitochondria in control cells (n = 328) and treated cells (n = 316). (**e**) The morphology of the mitochondria was assessed in the skin after the GTT test. Histological examination of the mitochondria from the skin of healthy and type I diabetes mice demonstrated abnormal morphology (red dotted circles) after glucose injection similar to that observed in the primary cells, while the majority of the mitochondria of the control mice in the healthy and type I diabetes groups were healthy in shape (yellow dotted circles). Scale bar = 1 µm. (**f**) Quantification of mitochondria in the skin. A significant increase in abnormal mitochondria was observed in the skin of both groups of healthy and type I diabetic mice injected with d-glucose (n = 42 cells for all groups except n = 35 cells for the type I diabetic mice injected with glucose) relative to their counterparts. One-way ANOVA multiple comparison was used for statistical analysis in (**b**) (*P < 0.03; **P < 0.004; ****P < 0.0001), (**d**) (**P < 0.001; ****P < 0.0001) and (**f**) (*P < 0.03; ***P < 0.0003; ****P < 0.0001).

To test the hypothesis that mitochondria act to buffer intracellular calcium levels through the action of the mitochondrial calcium uniporter complex^43,44^, thereby affecting mitochondrial metabolism and the morphology of the mitochondrial network^45,46^, we first examined the effect of the extracellular glucose concentration on mitochondrial morphology using electron microscopy (EM). The results showed that the majority of the mitochondria were subjected to a modification in their morphology when cells were incubated with 25 mM d-glucose during 45 min (Fig. 4a). For quantification purposes, we classified the mitochondria into two groups: 1—a normal group that can take various shapes (elongated or round) with arranged cristae and an electron-dense matrix (yellow dotted circles) (Fig. 4a), and 2—an abnormal group that are swollen, enlarged and round in shape, the disarrangement of their cristae accompanied by a partially or totally electron-lucent matrix (red dotted circles) (Fig. 4a).

Quantitative analyses indicated that while the majority of the mitochondria in the cells incubated with low glucose during 45 min belonged to the normal group, the majority of those in the cells incubated with high glucose exhibited an abnormal shape (Fig. 4a,b). Since mitochondria always behave as a network^47,48^, we then assessed the effect of a high glucose concentration on the morphology of the mitochondrial network at 45 min in keratinocyte mitochondria staining with the MitoTracker probe. While the majority of the control keratinocytes demonstrated an interconnected network located mostly around the nucleus, the majority of the cells incubated with high glucose exhibited a fragmented mitochondrial network that was dispersed in the cytoplasm (Fig. 4c,d).

To examine whether mitochondria are also affected under our in vivo conditions, skin biopsies were collected at 45 min post-injection from the four groups of mice and visualized under EM. A similar mitochondrial phenotype was observed in the basal keratinocytes of both the healthy and type I diabetic groups upon receiving an injection of glucose. Indeed, while the majority of the mitochondria in the placebo-injected healthy group belonged into our normal mitochondrial class (yellow dotted circles) (Fig. 4e,f), the majority of those in both the glucose-injected healthy and type I diabetic groups were swollen, enlarged and round in shape with disarrangement of their cristae (red dotted circles) accompanied by a partially or totally electron-lucent matrix (Fig. 4e,f).

Significantly, the number of mitochondria belonging to the normal group was lower in the placebo-injected type I diabetic mice than in the placebo-injected healthy mice and that of the abnormal group higher (Fig. 4f). This could have been due to the high glucose level found in the skin of the type I diabetic mice even in the absence of glucose injection (see Fig. 1c).

### Trisk 95 knockout abolishes the increase in intracellular calcium mediated by a high glucose concentration and rescues the mitochondrial phenotype in the skin and its primary keratinocytes

**Figure 5:**
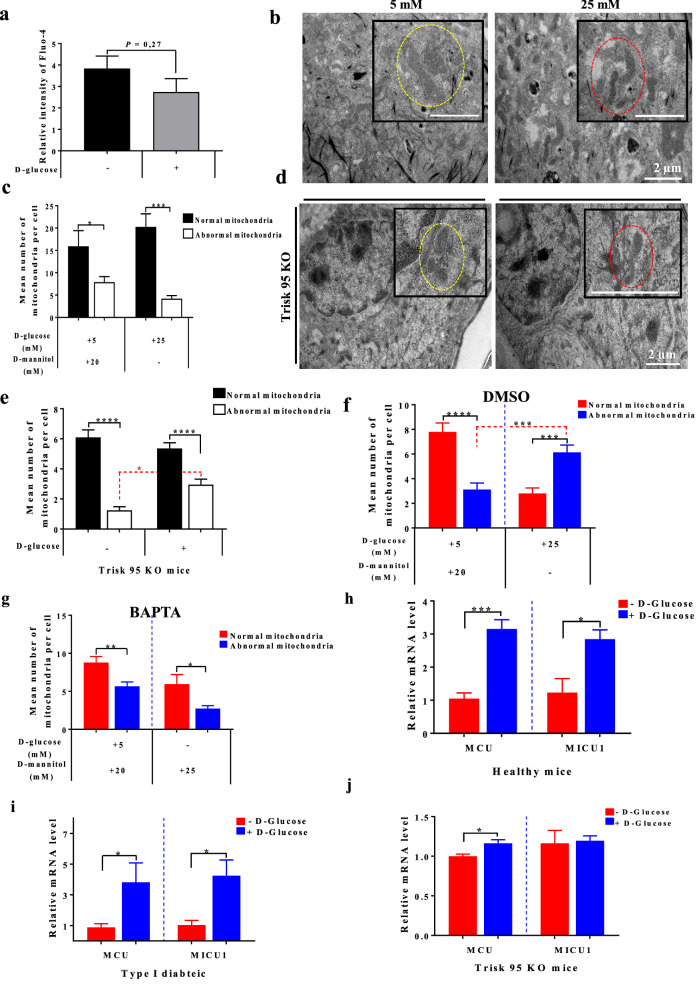
Trisk 95 KO rescues phenotype changes in both calcium and mitochondria in the skin and its primary cells. (**a**) Calcium uptake after high glucose injection was assessed. The relative intensity of Fluo-4 staining was measured by flow cytometry. The results are presented as means ± SEM (n = 3 injected with PBS and n = 4 injected with glucose and 10,000 cells/mouse). (**b**,**c**) High glucose did not affect the mitochondria phenotype in Trisk 95 KO keratinocytes. Keratinocytes isolated from Trisk KO exhibited a normal mitochondrial phenotype (**b**), scale bar = 2 µm. Quantification of mitochondria confirmed the histological observation (n = 20) compared with the controls (n = 29) (**c**). (**d**,**e**) High glucose did not affect the mitochondrial phenotype of Trisk 95 KO skin. The majority of the mitochondria from the Trisk 95 KO basal keratinocytes represented a normal phenotype (red dotted circles), as also observed in the controls (yellow dotted circles) (**d**), scale bar = 2 µm. Quantification of the mitochondria in the skin injected with d-glucose (n = 158 cells) as compared with the controls (n = 132 cells) (**e**). (**f**) DMSO pre-incubation did not affect the mitochondrial phenotype brought about by high glucose. (**g**) BAPTA pre-incubation rescued the mitochondrial phenotype induced by high glucose in primary keratinocytes. (**h**,**i**,**j**) Effect of high glucose on mitochondrial uniporter proteins in the skin. MCU and MICU1 expression in the skin. mRNA expression levels of *MCU* and *MICU1* in the skin of healthy (**h**), type I diabetic (**i**) and Trisk 95 KO (**j**) mice was examined using qRT-PCR. The results are shown as the averages after normalization to the controls ± SEM (n = 6/group). The two-tailed Student’s t-test was used in (**a**) (*ns* not significant *P* = 0.27), (**g**) (**P < 0.003, *P < 0.041), (**h**) (***P < 0.0002, *P < 0.012), (**i**) (*P < 0.042 and *P < 0.025) and (**j**) (*P < 0.035). One-way ANOVA multiple comparison was used in (**c**) (*P < 0.03; ***P < 0.0002), (**e**) (*P < 0.01; ****P < 0.0001), and (**f**) (****P < 0.0001; ***P < 0.0002; ***P < 0.0008).

To investigate the functional relation between Trisk 95 and intracellular calcium during the skin’s response to high blood glucose, skin biopsies were collected from the Trisk 95 KO mice 45 min after the injection of glucose or a placebo. To evaluate the basal calcium level, keratinocytes isolated from both groups were loaded with Fluo-4 AM. The results did not show any significant differences in basal calcium concentration between the placebo and glucose-injected Trisk 95 KO mice (2.74 ± 0.62 vs 3.83 ± 0.58, *p* = 0.27) (Fig. 5a), indicating that Trisk 95 expression is necessary for high blood glucose-mediated modification to the skin calcium homeostasis.

In order to assess the role of Trisk 95 in the transitional modification of the mitochondrial phenotype, keratinocytes isolated from Trisk 95 KO mice and cultivated for 45 min in the presence of low or high glucose were examined by EM. Interestingly, the majority of the mitochondria in the Trisk 95^−/−^ keratinocytes incubated with high glucose exhibited an elongated shape accompanied by arranged cristae and an electron-dense matrix (Fig. 5b,c), a phenotype that has been classified as normal. These results indicate that Trisk 95 expression plays an important role in keratinocyte responses to the extracellular glucose level. To examine this hypothesis further, skin mitochondrial phenotype comparisons were made between the placebo and glucose-injected Trisk 95^−/−^ mice, whereupon the results showed that the majority of the mitochondria in the basal keratinocytes were normal in both groups (Fig. 5d,e), indicating that Trisk 95 downregulation blocks the mitochondrial phenotype modification mediated by a high blood glucose concentration in.

To further examine the role of calcium in mitochondrial phenotype modification when monitoring high extracellular glucose concentrations, keratinocytes isolated from healthy mice were first incubated with BAPTA, a well-known calcium chelator, for 45 min and then subjected to low or high glucose conditions for 45 min. Histological examination of the mitochondrial phenotype indicated that treatment with DMSO (in which BAPTA was dissolved) did not affect the influence of extracellular glucose on the mitochondrial phenotype (Fig. 5f, see also Fig. 4b). The addition of BAPTA, however, significantly reduced the number of abnormal mitochondria in the cells subjected to high glucose (Fig. 5g). Taken together, our results indicate that the mitochondrial phenotype is triggered by calcium changes and can be rescued by adding BAPTA.

**Figure 6:**
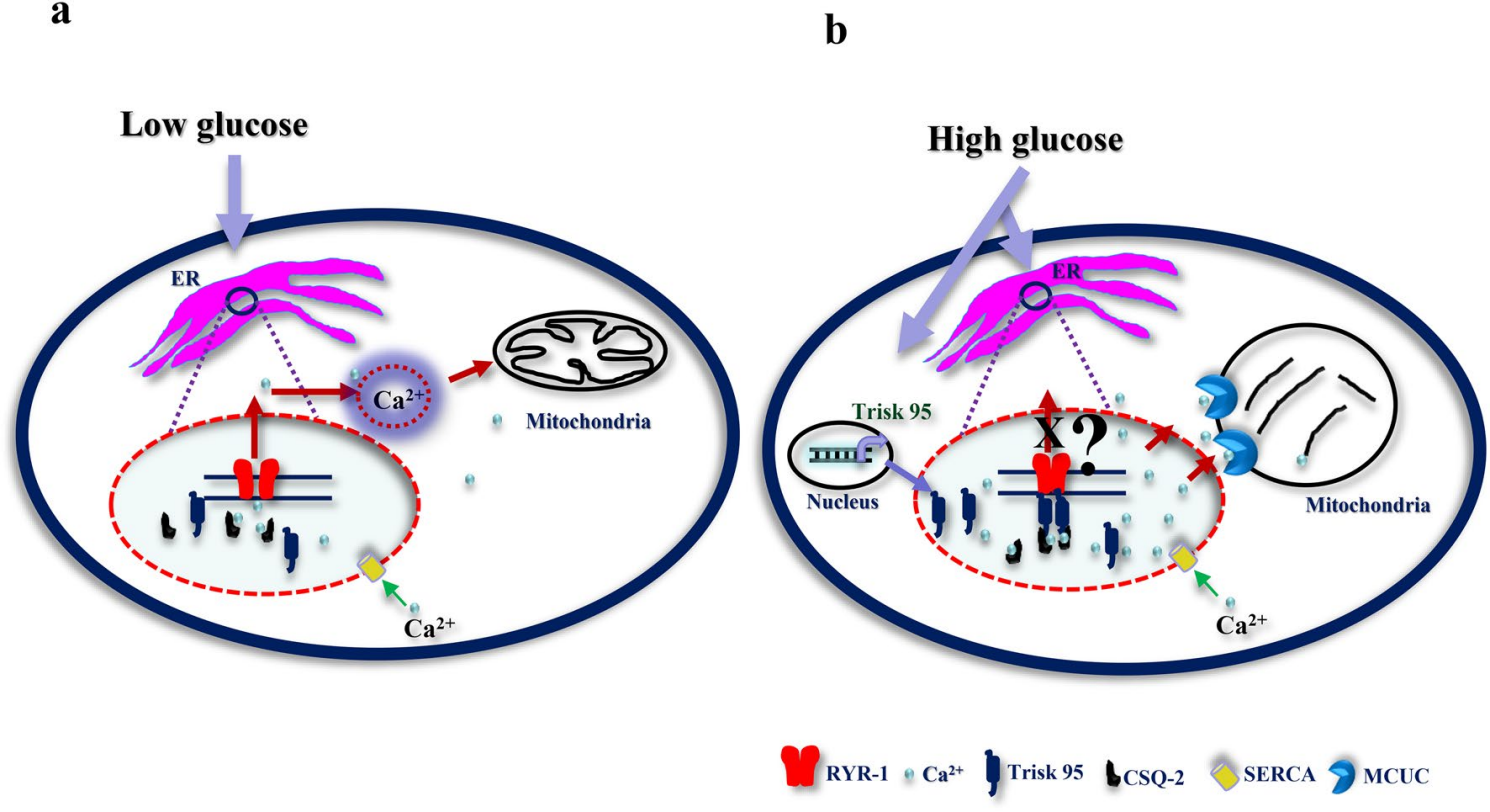
Proposed model for the mechanism by which high glucose triggers calcium uptake in the ER and mitochondria via Trisk 95. Under normal conditions (low glucose) no binding occurs between Trisk 95 and RYR and the calcium level in the ER is thus maintained via constant exchange with the cytosol. The calcium level is important for the functioning of the mitochondria (**a**). In the presence of high glucose Trisk 95 is overexpressed at transcriptional and then protein level, leading to its binding to the RYR channel and resulting in the closure of the latter. As a consequence, the increased calcium store in the ER triggers mitochondrial changes because of calcium uptake via MCUC (**b**).

In order to analyse the molecular mechanism by which cytoplasmic calcium fluctuation could trigger mitochondrial phenotype alternation in the skin and its primary cells, we examined at 45 min the expression of MCU and MICU1 (two well-known mitochondrial calcium uniporters), and found that, upon subjection to high glucose injection, *MCU* and *MICU1* expression at the mRNA level were significantly increased in both healthy (3 and 2.8-fold increases, respectively) and type I diabetic mice (3.8 and 4-fold increases) compared with their control counterparts (Fig. 5h,i). Interestingly, when looking at MCU and MICU-1 expression levels in the Trisk 95 KO mice, we found that only *MCU* was significantly upregulated in the glucose-injected mice relative to the placebo-injected ones (a 1.16-fold increase) but not *MICU1* (Fig. 6j). These findings suggest firstly that MCU and MCU1 are required together for activation of the mitochondrial calcium uniporter complex in keratinocytes, allowing calcium uptake, and secondly that the main function of Trisk 95 in keratinocyte responses to high extracellular glucose is to orchestrate that activation.

## Discussion

This report provides evidence of skin functionality and its ability to sense internal changes communicated from other organs, and as an example; we describe a novel paradigm of using skin cells as biological sensors for mirroring blood glucose levels (Supplementary Fig. 5). Our hypothesis in this schematic diagram is that the blood sugar level systemically influences the skin, resulting in the activation of specific mediators that can be used as potential targets when applying genome-editing technology. Thus, the edited stem cells are turned_on or _off by visualisation of the induced action of sugar. This strategy may be extended to monitor other health parameters in normal and diseases conditions and it is this that represents the main goal of our registered patent (No. PCT FI2016/050917, https://encrypted.google.com/patents/WO2017109292A1?cl=no).

As the first proof of this concept, our results show that upon an increase in blood glucose concentration the skin in both healthy and type I diabetic mice takes up more glucose, suggesting that this takes place through an insulin-independent pathway. When looking for the mechanism underlying this increased uptake of glucose by keratinocytes we found that *Glut-1* mRNA expression was significantly increased in the mice receiving d-glucose compared with the controls, and that *Glut-1* mRNA expression was significantly higher in the type I diabetic mice than in the healthy mice, which may explain the higher skin glucose level. However, the (non significant) decrease in *Glut-1* mRNA expression and protein levels observed in the d-glucose-injected type I diabetic mice compared to their controls may be explained by an auto-regulation of the glucose uptake via reduction in the expression of Glut-1, as previously shown^24^.

In view of our results showing that a high extracellular glucose concentration induces Trisk 95 expression at the protein as well as the transcriptional level, we propose Trisk 95 as a new biomarker for glucose in keratinocytes. This result is a promising one obtained with our mouse model in which the injection of an edited Trisk 95-iPSC was shown to be feasible. Nevertheless, because of the ethical restriction on performing the same manipulation on humans, we thought that it was worth investigating the potential role of Trisk 95 in glucose signalling in keratinocytes in order to find the molecular signature in terms of outcomes extending beyond this signalling.

Triadin is a key player in the functioning of the heart and skeletal muscles in that it regulates the excitation–contraction (EC) coupling process^41,49–51^. Moreover, it modulates intracellular calcium haemostasis via its interaction with various proteins such as Junctin^42^, RYR^52,53^ and CSQ^54^. Indeed, it has been shown that Triadin regulates calcium release from the sarcoplasmic reticulum (SR) by binding directly to the RYR receptor in a specific domain^55^.

In view of its collaboration mechanism with RYR and CSQ, we testes whether their expression is modified under our conditions and looked at their expression in our mouse models. The results showed a significant increase in the *RYR-1* mRNA level in both glucose-injected healthy and type I diabetic mice relative to their counterparts. Regarding *CSQ-2* mRNA, its expression level was significantly increased in the glucose-injected type I diabetic mice (Supplementary Fig. 4a,b). Similar results were obtained from primary keratinocytes after 45 min of high glucose incubation (Supplementary Fig. 4c). On the other hand, the *RYR-1* and *CSQ-2* mRNA levels in Trisk 95 KO skin did not change in response to a high-glucose injection (Supplementary Fig. 4d). Combining our findings with others that demonstrate the essential role of Trisk 95 in muscle function, we propose a similar model for the mechanism by which Trisk 95 may play a potential role in orchestrating calcium signalling as a consequence to fluctuations in blood glucose concentration. In healthy individuals (blood glucose level ~ 5 mmol/l), Trisk 95 does not bind RYR-1 or CSQ-2 and a constant calcium leak from ER into the cytosol is maintained via the RYR-1 channels. Under those conditions, the cytoplasmic calcium is involved in physiological processes in the mitochondria. Under type I diabetic conditions, however, in which blood glucose levels are high (≥ 15 mmol/l), Trisk 95 is upregulated at both levels (mRNA and protein). Binding of the skin Trisk 95/CSQ-2 complex to the RYR-1 channels leads to their closure and subsequently to increased ER calcium stores. This increase in ER calcium eventually triggers modifications in the mitochondrial network and consequently in its optimal functioning. It is worth mentioning here that deeper investigations are needed to demonstrate the relationship between Trisk 95 and RYR channel activity.

Given that calcium signalling is a key regulator of mitochondrial behaviour^56^, we hypothesize that an increase in intracellular and ER calcium may affect mitochondrial function. Our data obtained in vivo and in vitro indicate that mitochondria present a specific phenotype after high glucose in the skin of both healthy and type I diabetic mice. The mitochondrial phenotype could be attributable to calcium uptake resulting in mitochondrial damage^57^. The mitochondrial calcium uniporter complex is responsible for calcium uptake across the inner membrane^43,44,58,59^, and in agreement with this, our data showed that the expression of *MCU* and *MICU1* at the mRNA level was significantly upregulated after high glucose in both healthy and diabetic mice.

Overall, our study highlights the rapid, dynamic physiological behaviour occurring in the skin and its primary keratinocytes in response to changes in blood glucose concentration. The data demonstrate for the first time that high blood glucose for 45 min induces the activation of a number of players that are cross-talking, leading to the activation of signalling cascades. Trisk 95 is shown to be an essential mediator in the interaction between these cascades and we proposes that the ensuing calcium or its mitochondrial metabolites can be used as indicators of blood glucose level in the skin.

## Materials and methods

The animal care and experimental procedures in this study were in accordance with Finnish national legislation on the use of laboratory animals, the European Convention for the protection of vertebrate animal used for experimental and other scientific purposes (ETS 123), and the EU Directive 86/609/EEC. The animal experimentation was also authorized by the Finnish National Animal Experiment Board (ELLA) as being compliant with the EU guidelines for animal research and welfare.

### Cell culture and glucose treatment

Primary keratinocytes were isolated from the skin of 6–8-week-old mice as previously described (Supplementary ref. 1). Briefly, mice (C57/BL6) were sacrificed (by cervical dislocation) and the hair shaved from their backs. The skin was then sterilized by immersion in a beaker of 10% betadine for 2 min, in a beaker of 70% ethanol for 1 min and then in sterile Dulbecco’s phosphate (PBS) for 1 min. The skin samples were treated with trypsin for 2 h at 33 °C to separate the epidermis from the dermis. Keratinocytes were cultured in F12 (21765-037)–DMEM (41965-062), medium, both from Thermo Fisher, supplemented with hydrocortisone (0.5 µg/ml), epidermal growth factor (EGF) and insulin (5 µg/ml), all from Sigma. The medium was changed three times a week. When the cultures reached 70–80% confluence the keratinocytes were incubated with two concentrations of d-glucose (G7021-Sigma) (5 mM + 20 mM mannitol (for osmolality)) or 25 mM for 45 min and then used for the various experiments.

### The type I diabetic mouse model

Type I diabetes was induced after one single intraperitoneal (IP) injection of streptozotocin (STZ), as previously described^36^. One week before the experiment, all the mice were housed in individual cages. On the day of the experiment, they fasted for 4 h and were then divided into two groups: controls and type I diabetic mice. The control mice were injected IP with Na citrate buffer (pH 4.5), while the type I diabetic mice were injected with STZ (150 mg/kg) diluted in Na citrate buffer to a total volume of 100 µl. The mice were diagnosed as diabetic when their blood glucose level was ≥ 15 mmol/l.

### Insulin measurement

To determine the insulin concentration in the serum, blood samples were collected from the facial vein of the mice 45 min after the d-glucose injection. Serum was obtained after centrifugation at 3,500 rpm for 10 min at 4 °C and insulin was measured using the Insulin Rodent (Mouse/Rat) Chemiluminescence ELISA kit (ALPCO) according to the manufacturer’s instructions.

### Glucose tolerance test

For adaptation purposes, the mice were housed in individual cages for 1 week. Prior to the experiment the healthy and type I diabetic mice fasted overnight (12 h) and each group was then divided into two parts, yielding four groups in all: healthy and type I diabetic groups that were injected intraperitoneally (IP) with PBS, and healthy and type I diabetic groups that were injected IP with d-glucose (2 g/kg). Blood glucose levels were monitored at 0, 5, 15, 30- and 45-min post-injection. After 45 min, all the mice were killed and skin samples were collected for the various experiments (RT-qPCR, western blotting and EM).

All the animal procedures were performed in accordance with the European Convention (ETS 123), EU Directive 86/609/EEC and the Finnish national legislation, as approved by the local ethical committee.

### Luciferase reporter assay

The *Triadin* promoter region containing a 882 bp (chr6:123636957 + 123637838) DNA fragment was cloned into the pGL3 basic vector (Promega) upstream of the luciferase reporter gene. HaCat cells were seeded in a 96-well white plate at a concentration of 2 × 10^4^ in the presence of 100 μl DMEM (41965039 gibco) medium supplemented with 10% FBS and 1% PS. 12 h later, the medium was replaced with 100 µl of DMEM-free serum supplemented with 1% PS containing 5 mM d-glucose + 20 mM d-mannitol or 25 mM d-glucose (Sigma). The cells were then immediately transfected with a promoter construct, negative and positive control plasmids (100 ng each) and an internal control plasmid pGL4.74 (40 ng) (Promega), using Turbofect (0.533 Thermofischer Scientific) as the transfection reagent according to the manufacturer´s protocol. The cells were incubated at 37 °C in 5% CO. Two days later the Dual-Glo Luciferase Assay System (E2940, Promega) was used to measure the luciferase activity of the cells on a bio-luminometer, following the manufacturer´s protocol.

To avoid interference from firefly luminescence, all the samples on the 96-well plate were measured before inducing renilla luminescence. All the readings were based on measurements obtained from at least three replicate wells. The change in the activity of each gene promotor was determined by measuring the activity of luciferase in the transduced keratinocytes incubated with high glucose (25 mM) versus those incubated with low glucose (5 mM). Statistical significance was analysed using the two-tailed Student’s t test. Each experiment was performed independently three times. The following primers were used for cloning the Triadin promoter region (882 bp):

Triadin-882-NheI-F: CTAGCTAGCTTCTCCCCCTGGAAACAACC
Triadin-882-XhoI-R: GTACTCGAGCACCTGTCTAGGAGTGG.

### Proteomic analysis

Details of the sample preparation, nano-scale liquid chromatographic tandem mass spectrometry analysis, database search, processing of the results and label-free quantitative data analysis are provided in the “Supplementary information” section.

### Western blotting

Equal amounts of total protein were resolved by SDS-PAGE and electrophoretically transferred to nitrocellulose membranes (BioTop 741,280). The membranes were then incubated overnight at 4 °C with a 1:1,000 dilution of anti- Trisk 95 (Supplementary ref. 2), Glut-1 (Supplementary ref. 3) 1:5,000 anti-GAPDH (MAB374, Millipore), 1:5,000 anti-β-actin (A5441, Sigma Aldrich). After treatment with peroxidase-linked antibody (Dako, polyclonal) for 1 h, blots were developed using the 20X LumiGLO® Reagent and 20 × Peroxide (Cell Signaling Technology, #7003).

### Measurement of glucose uptake and intracellular glucose consumption

To evaluate glucose uptake or its intracellular concentration, we used the YSI 2950 Biochemistry Analyzer (YSI Life Sciences), which employs immobilized enzyme to catalyse the corresponding chemical reactions in order to measure the concentration of a specific metabolite. Briefly, to measure glucose uptake, keratinocytes were incubated in triplicate for 45 min in a 24-well plate in 1 ml of DMEM complete medium supplemented with glucose (5 mM or 25 mM). The concentration of glucose in the collected medium was evaluated by the YSI analyser using corresponding fresh medium as a reference.

To measure intracellular glucose concentrations, skin samples were collected at different time-points after a glucose injection from the various groups of mice and then immersed in a hypotonic buffer (2.5 mM Tris/HCl, pH 7.5 and 2.5 mM MgCl_2_) on ice for 15 min, homogenized and then sonicated for 15 s. The concentrations of glucose in the skin lysates were then evaluated. The data were expressed as mmol·μg^−1^ of protein.

### Intracellular calcium imaging and measurement

To image and measure intracellular calcium changes in vivo, primary keratinocytes were isolated and cultured in dishes with a glass bottom (35/10 MM, Greiner Bio-one International, 627,871) for 1 week. On the day of the experiment the cells were washed and incubated for 45 min in the presence of 5 mM d-glucose + 20 mM mannitol or 25 mM d-glucose. Then Fluo-4 AM (2 µM final concentration) (Thermo Fisher, F14201) was added directly to the medium and incubated for another 30 min at 37 °C. Changes in the intracellular calcium level was calculated by measuring the background-subtracted fluorescence signals (F1) and dividing these by the baseline signal (F0) before adding thapsigargin (Sigma, T9033) to a final concentration 100 nM. Zeiss Cell Observer Microscope (Carl Zeiss, Germany) in epifluorescence imaging mode was used to detect and measure the fluorescence of Fluo-4. The data were analysed and calculated by using Zen 2012 (Blue), the 2018 version of Origin and presented as means ± standard deviation.

To assess calcium levels after GTT, keratinocytes were isolated from skin biopsies collected at 45 min and stained Fluo-4 AM was added to the medium and incubated in the dark for 30 min. After two washes with PBS, the cells were analysed by flow cytometry. Data from ten thousand individual cells were collected for each sample.

### Immunostaining of cells

To examine the expression of Trisk 95 and its localization in the primary keratinocytes, cells were cultured in dishes with a glass bottom for 45 min after treatment with two concentrations of glucose. They were then fixed in 4% paraformaldehyde (PFA) for 10 min at RT and incubated with blocking solution (10% BSA, TBST (Tris buffer saline-Tween) 0.1%) for 30 min. This was followed by a further incubation with the primary antibody anti-Trisk 95, as previously described (Supplementary ref. 2), diluted in the blocking solution for 1 h at RT and then with DAPI, and with a secondary antibody for 30 min. Fluorescence was detected using a confocal microscope (Zeiss LSM 780, Carl Zeiss, Germany).

### Transmission electron microscopy (TEM)

Skin biopsies were collected at 45 min post-injection from the four groups (three mice per group) after the GTT assay, and cultured cells were mounted on coverslips and fixed in 1% glutaraldehyde and 4% formaldehyde in 0.1 M phosphate buffer, pH 7.4. The remaining steps were followed as previously described (Supplementary ref. 4). Sections from the skin samples and cells were examined using a Tecnai Spirit G2 transmission electron microscope. Images were captured with a Veleta CCD camera and Item software (Olympus Soft Imaging Solutions GMBH, Munster, Germany).

### Statistical analysis

GraphPad Prism software, version 7, was used for the statistical analyses. The one-way or two-way ANOVA test or the two-tailed Student t-test were employed and *P-values less than 0.05 were considered significant.

## Supplementary information

Supplementary Information.

## Data Availability

The data sets generated and/or analyzed during the current study are available from the corresponding author on request.
