# Supplementary material for: Trisk 95 as a novel skin mirror for normal and diabetic systemic glucose level
Source: Sci Rep. 2020 Jul 22;10:12246. doi: 10.1038/s41598-020-68972-6 (PMC7376074; doi:10.1038/s41598-020-68972-6)
Supplement: Supplementary file 1 — Supplementary Information. [file 41598_2020_68972_MOESM1_ESM.pdf]

Trisk 95 as a Novel Skin Mirror for Normal and Diabetic Systemic Glucose Level

Nsrein Ali [1, 6], Hamid Reza Rezvani [2, 3], Diana Motei [1], Sufyan Suleman [1], Walid Mahfouf [2], Isabelle Marty [4], Veli-Pekka Ronkainen [5], Seppo J. Vainio [1, 6, 7]

[1] Faculty of Biochemistry and Molecular Medicine, Biocenter Oulu, Laboratory of Developmental Biology, University of Oulu, Oulu, 90220, Finland. [2] University of Bordeaux, Inserm, BMGIC, UMR 1035, Bordeaux, France. [3] Centre de Référence pour les Maladies Rares de la Peau, CHU de Bordeaux, Bordeaux, France. [4] Grenoble Institut des Neurosciences, University Grenoble, Inserm U1216, La Tronche, France. [5] Biocenter Oulu, [6] Infotech Oulu, University of Oulu, Oulu, 90220, Finland. [7] Borealis Biobank of Northern Finland, Oulu University Hospital, Oulu, Finland.

To whom correspondence should be addressed. E-mail: nsrein.ali@oulu.fi

Address for Correspondence:

Nsrein Ali, PhD
Faculty of Biochemistry and Molecular Medicine
Biocenter Oulu
Laboratory of Developmental Biology
Aapistie 5A, FIN-90220
Oulu University, Oulu, Finland
Telephone: +358-41 493 1342

Supplementary Figures

Supplementary Fig. 1

a

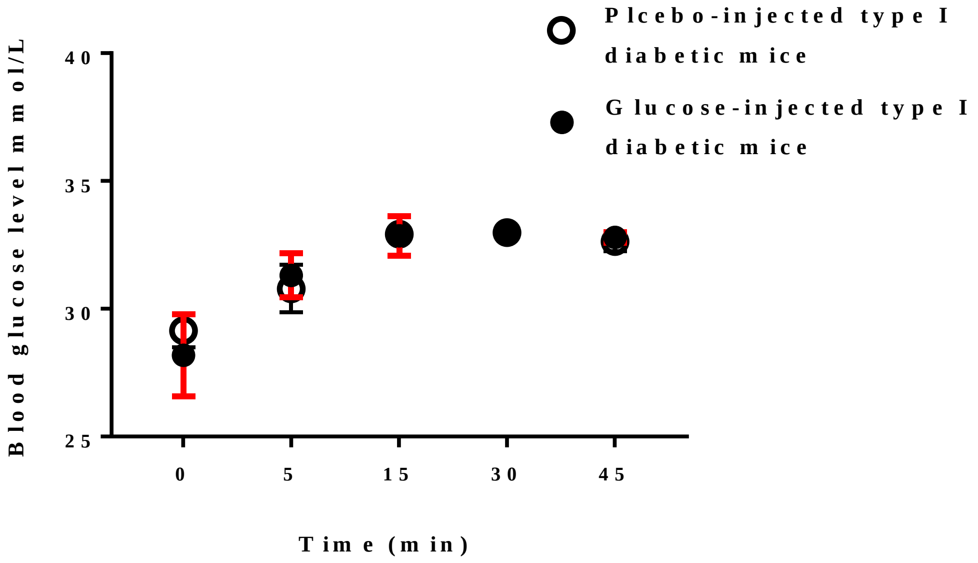

b

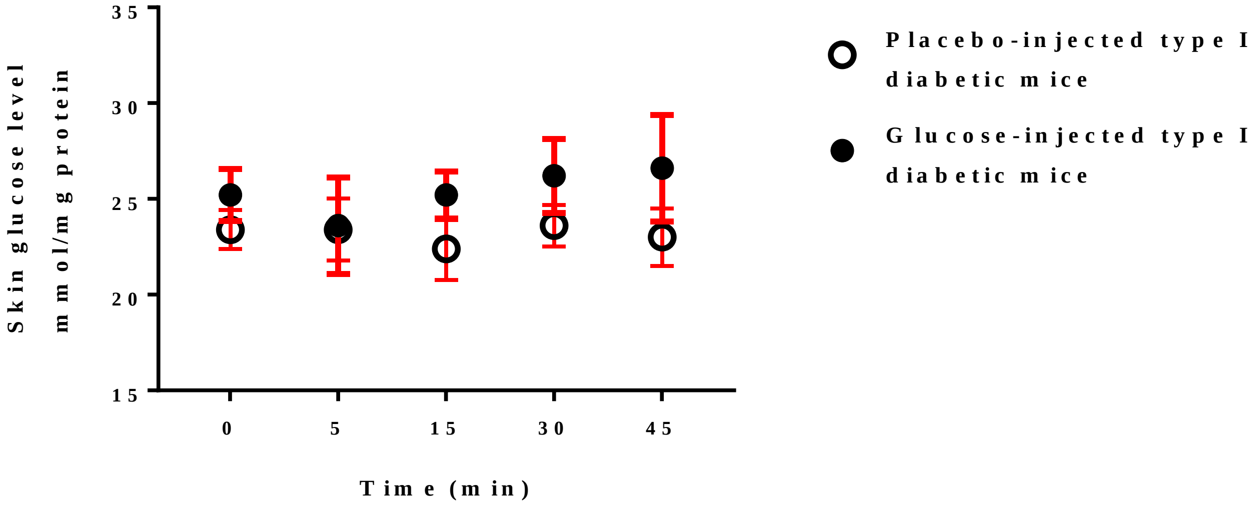

c

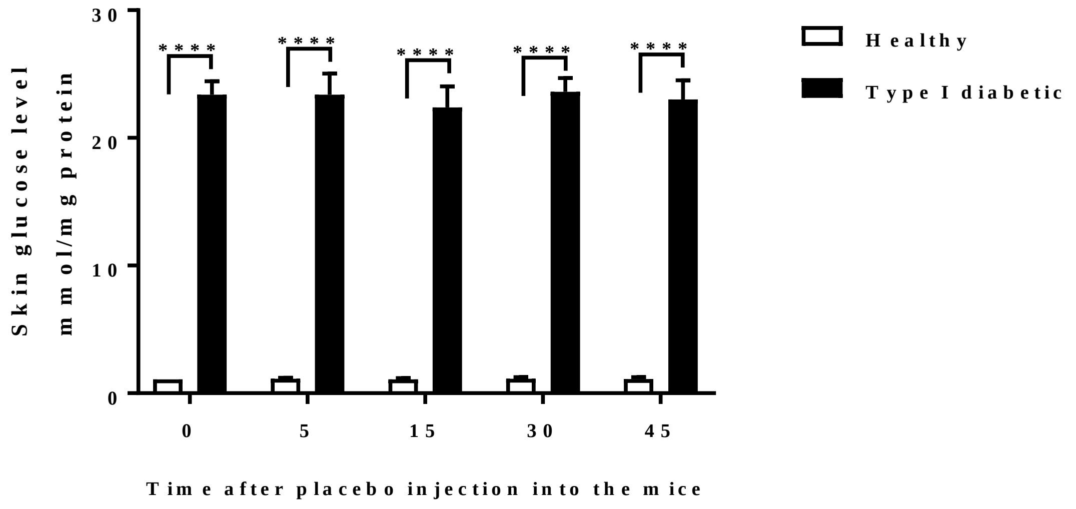
Skin glucose level
mmol/mg protein
30
20
10
0
****
0
5
15
30
45
Time after placebo injection into the mice
Healthy
Type I diabetic

d

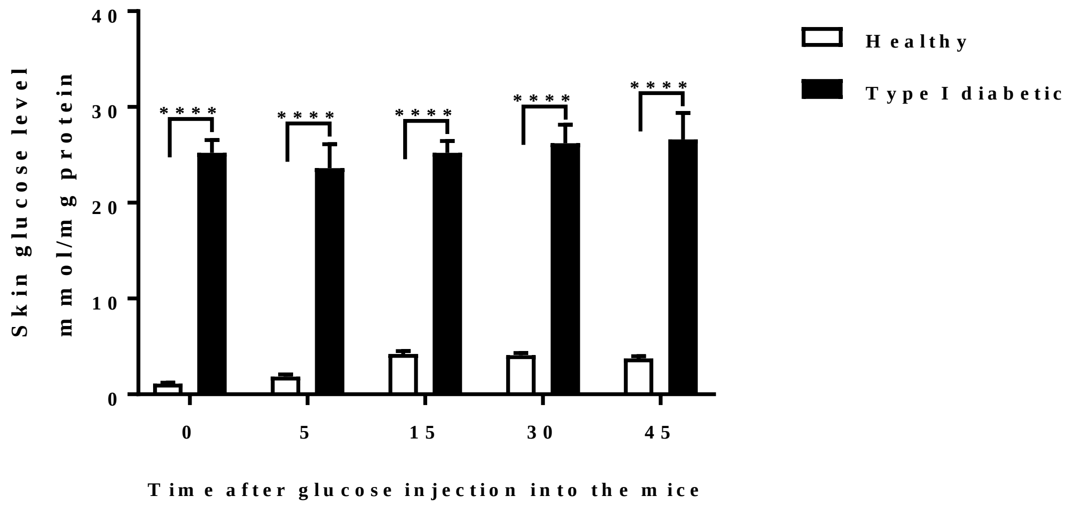
Skin glucose level
mmol/mg protein
40
30
20
10
0
****
0
5
15
30
45
Time after glucose injection into the mice
Healthy
Type I diabetic

e

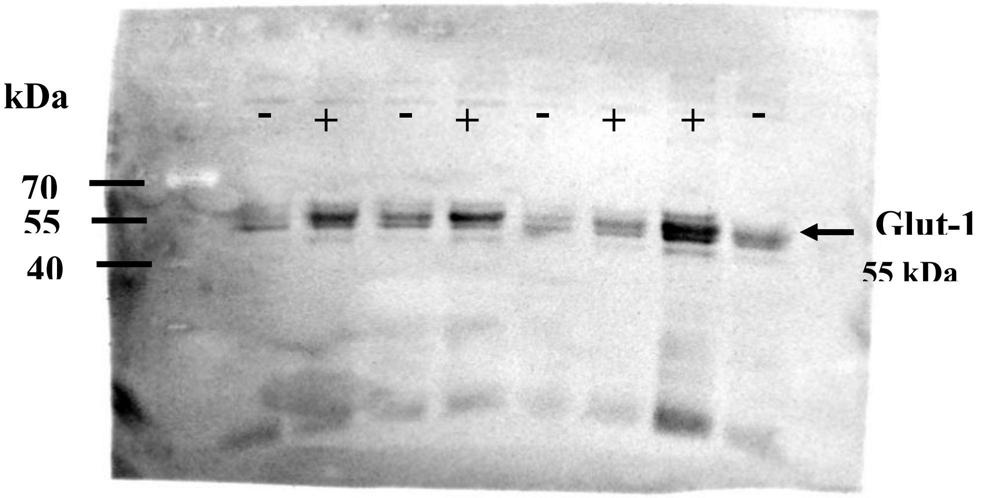
kDa
- + - + - + + -
70
55
40
Glut-1
55 kDa

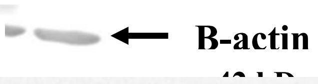
B-actin

f

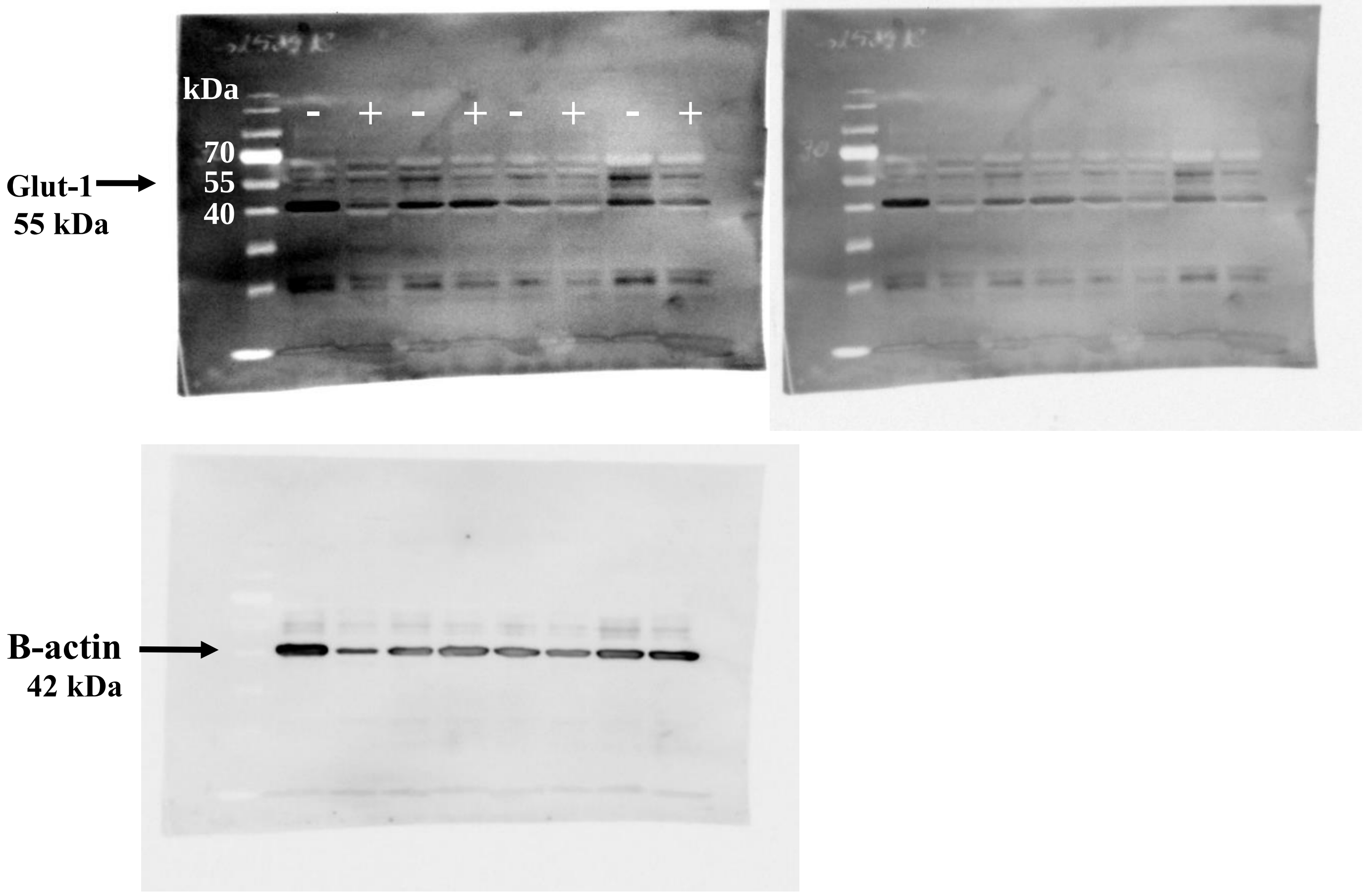
kDa
- + - + - + - +
70
55
40
Glut-1
55 kDa
B-actin
42 kDa

Supplementary Fig 1. Skin senses the modification in the blood glucose level. **a** and **b.** GTT assay using type I diabetic mice. Glucose levels were monitored in the blood (**a**) and the skin (**b**) at the given time points (n=5 mice/group). **c and d. S**kin glucose levels *in vivo*. The GTT assay was performed and skin biopsies were collected at 0, 5, 15, 30 and 45 min post-injection from the four groups (n=7 healthy, n=5 type I diabetic). Glucose levels in the skin were measured and normalized to the total concentration of proteins (mmol/mg protein). Skin glucose level comparisons between placebo-healthy and type I diabetic (**c**), then between glucose-injected healthy and type I diabetic mice (**d**) were performed. **e.** Uncropped blot of Glut-1 protein expression level in the skin of healthy mice. The mice were divided into two groups, the first (that is control) received PBS injection (-) and the second (that is treated) received glucose injection (+). The band observed with molecular weight of 55 kDa indicate the Glut-1, and □-actin (42 kDa) was used for loading control. **f.** Uncropped blot of Glut-1 protein expression level in the skin of type I diabetic mice. The mice were divided into two groups, the first (4 mice) received PBS injection (-) and the second (4 mice) received glucose injection (+). The band observed with molecular weight of 55 kDa indicate the Glut-1, and □-actin (42 kDa) was used for loading control.

Supplementary Fig. 2

a

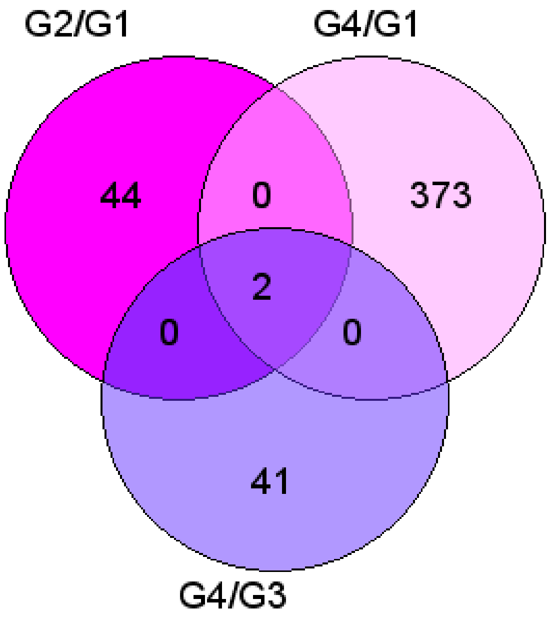

b

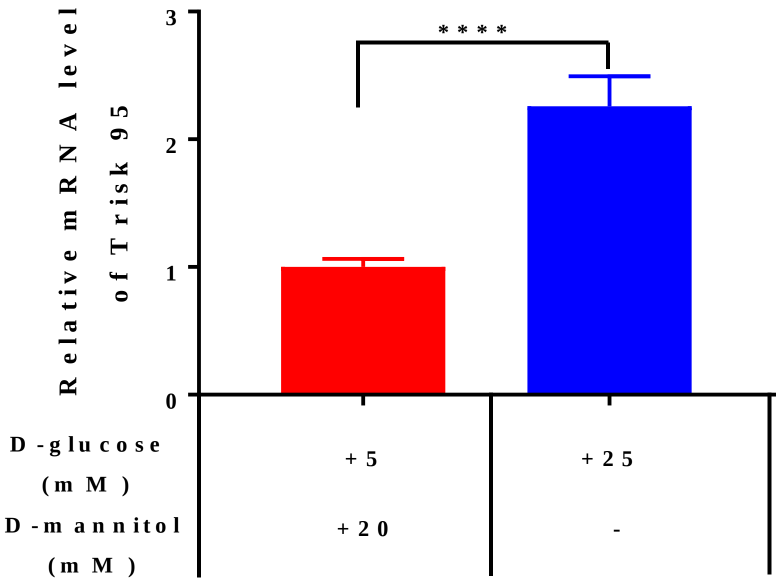

c

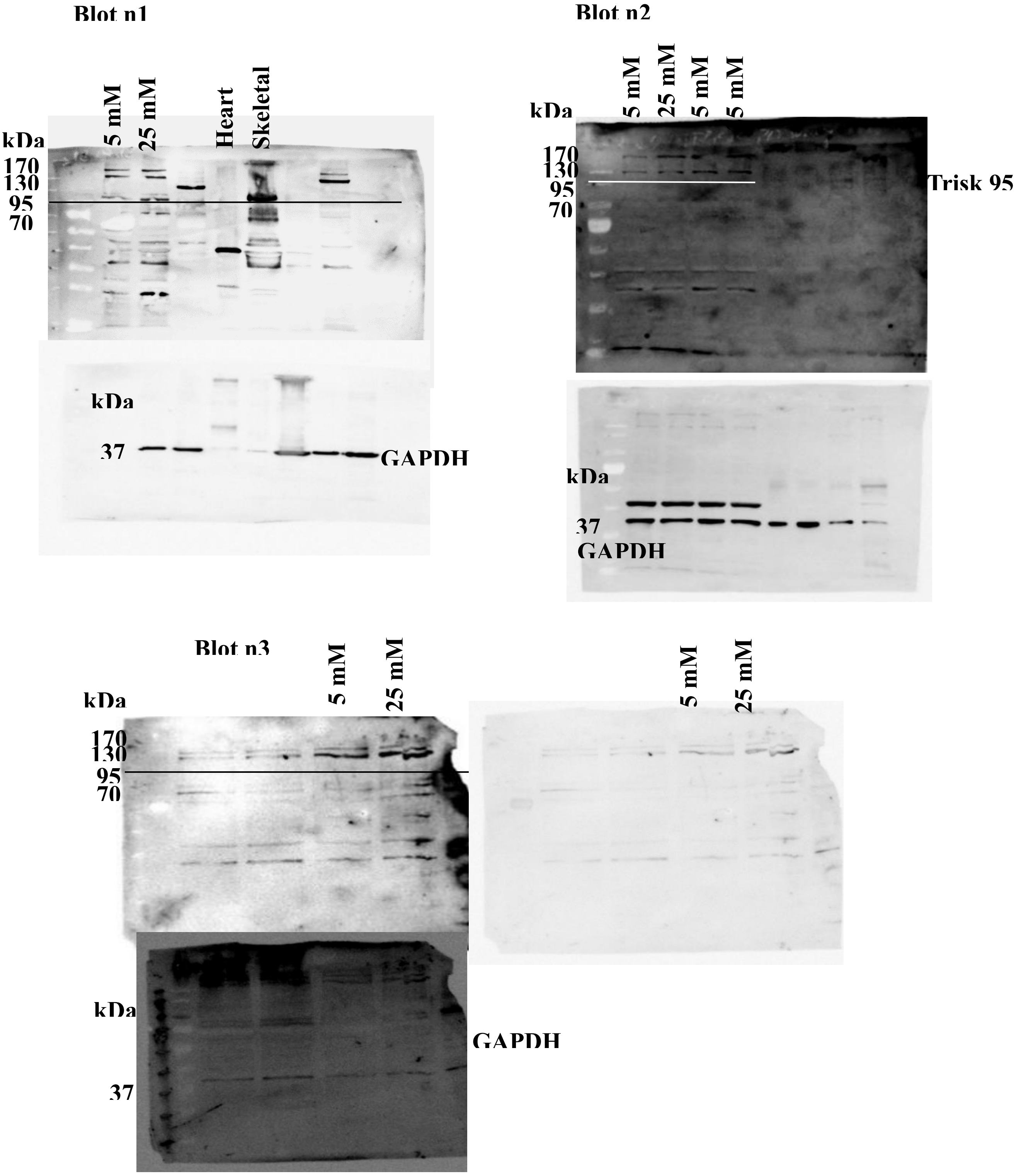

Blot n1
5 mM
25 mM
Heart
Skeletal
kDa
170
130
95
70
kDa
37
GAPDH
Blot n2
kDa
5 mM
25 mM
5 mM
5 mM
170
130
95
70
Trisk 95
kDa
37
GAPDH
Blot n3
5 mM
25 mM
kDa
170
130
95
70
5 mM
25 mM
kDa
GAPDH
37

Supplementary Fig 2. Triadin expression in the skin from type I diabetic mice. **a** Proteomic profile of the skin after glucose injection in vivo. G1 and G4 represent the healthy and type I diabetic groups injected with PBS, respectively. G2 and G3 represent the healthy and type I diabetic groups injected with D-glucose, respectively. **b** *Trisk 95* mRNA expression were quantified using qRT-PCR in primary keratinocytes at 45 min after incubation of medium containing high glucose concentration. The results are shown as averages after normalization to the control ± SEM in three separate experiments. **c** Uncropped blot of Trisk 95 expression in primary keratinocytes (lanes 1 and 2), in skeletal muscle (lane 3, as positive control), and in heart muscle (lane 4, as negative control). **c** Uncropped blots (n1, 2 and 3) of Trisk 95 expression from three independent experiments in which primary keratinocytes were incubated in presence of 5 or 25 mM D-glucose during 45 min. The band of ~ 95 kDa indicating Trisk 95 was used for quantification and normalized to GAPDH expression level from the three different experiments. For statistical analysis, two tailed Student's t-test was used in b (**** *P* <0.0001).

Supplementary Fig. 3

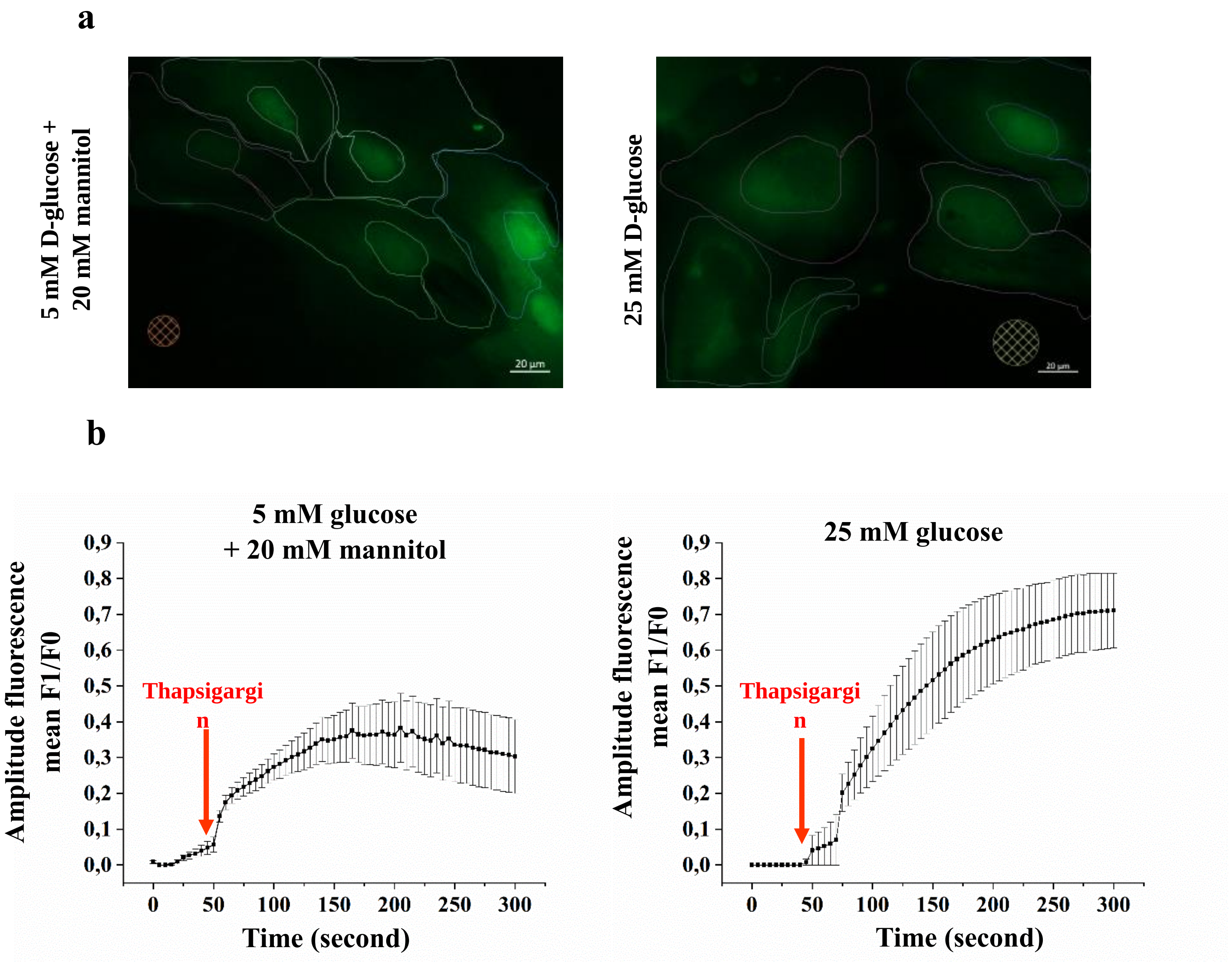

Supplementary Fig 3. Calcium release in primary keratinocytes. Cells were incubated with low or high glucose for 45 min, and then stained with Fluo-4 AM for 30 min. Live-cell time lapse imaging was used to evaluate calcium release from the ER after blocking SERCA with thapsigargin. **Two movies of keratinocytes** incubated with 5 mM D-glucose and 25 mM D-glucose, showing the Fluo-4 intensity changes after adding thapsigargin at 50 seconds. **b** Averaged fluorescence intensity curves in the cells incubated with low or high glucose before and after thapsigargin stimulation. The cytosolic fluorescence intensity was measured within the regions of interest excluding the nuclei in each cell. The maximum fluorescence amplitude and the increase in speed (of reaching half maximum amplitude) was measured under both sets of conditions.

Supplementary Fig. 4

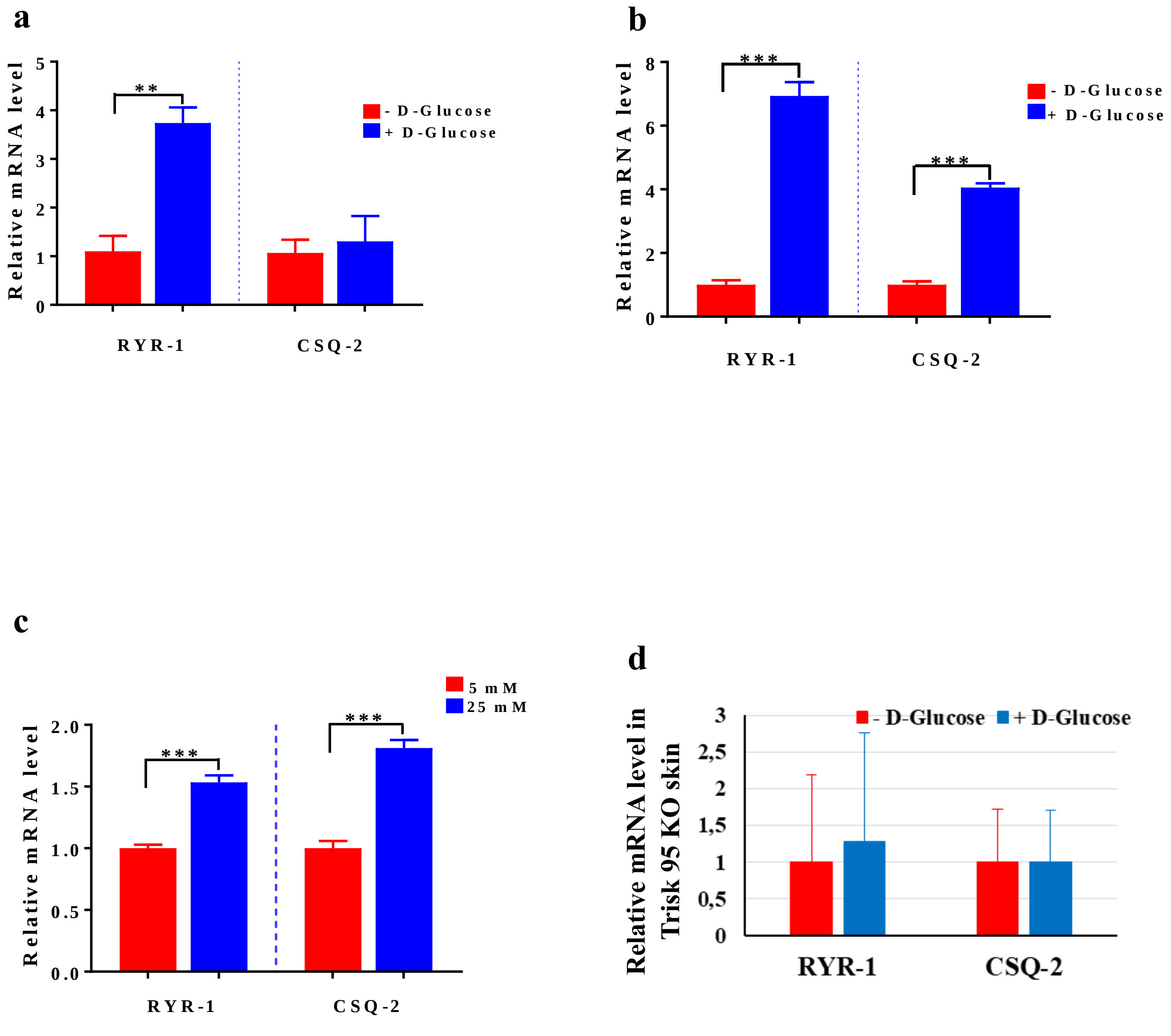

Supplementary Fig 4. High glucose induces changes in RYR-1 and CSQ-2 expression, while Trisk 95 silencing will rescue their expression. **a, b** and **c** RYR-1 and CSQ-2 expression in the skin and primary keratinocytes. mRNA expression levels of *RYR-1* and *CSQ-2* in healthy mice (a), type I diabetic mice (b) and primary keratinocytes (c). **d** RYR-1 and CSQ-2 expression in Trisk 95 KO mice. The results are shown as averages after normalization to the controls ± SEM (n=6/group of healthy mice, n=3/group of type I diabetic mice, and three independent experiments for the primary keratinocytes. Also, n=3 (Trisk 95 KO mice injected with PBS) and n=4 (Trisk 95 KO mice injected with glucose). The two-tailed Student's t-test was used for statistical analysis in a (** P <0.004, ns. not significant 0.70), b (*** P <0.001), c (*** P <0.001) and d (ns. not significant 0.2).

Supplementary Fig. 5

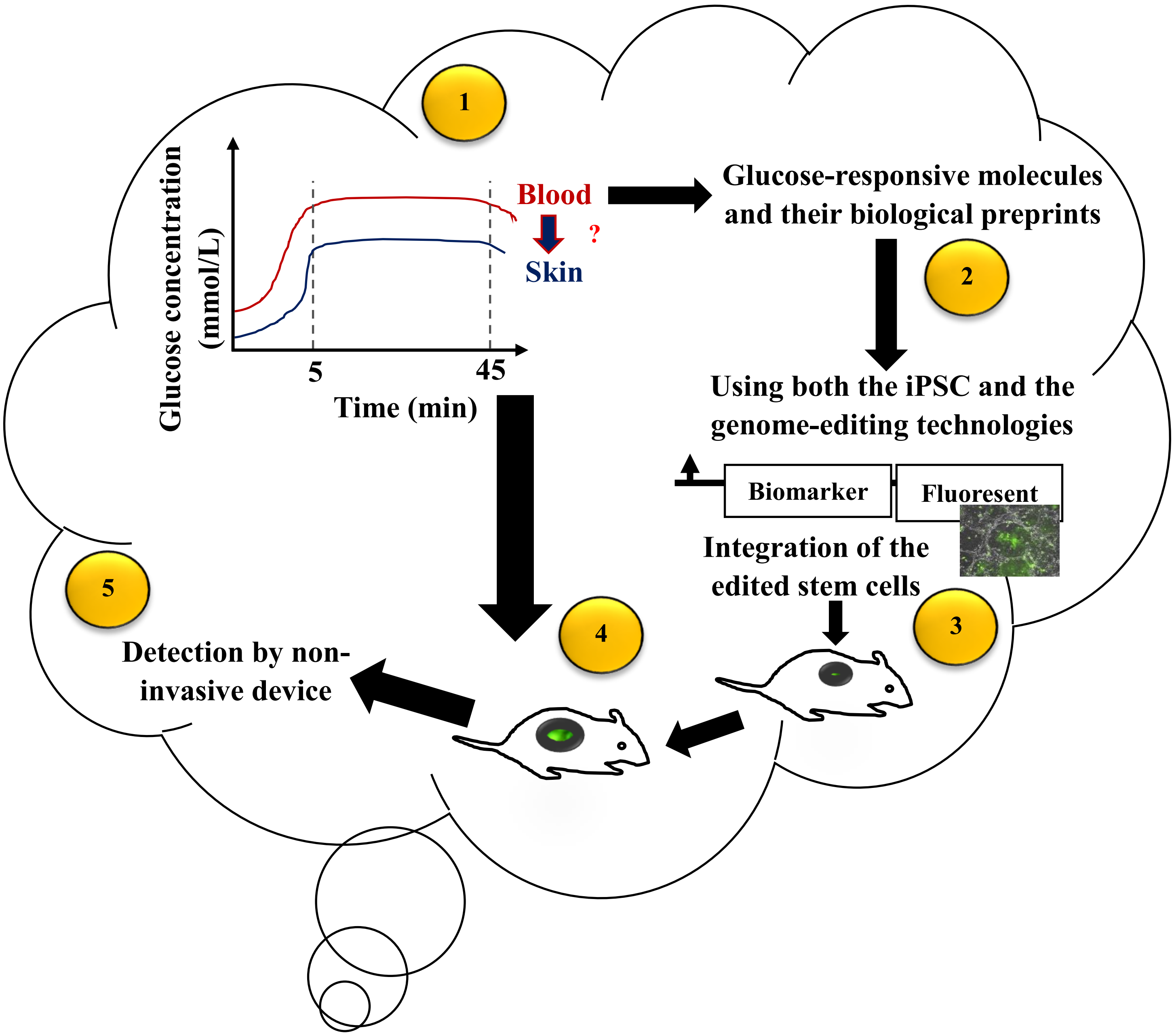

Supplementary Fig 5. Carton schema representing the different steps for developing a non-invasive glucose biosensor using mouse as model. The first step consists to monitor the blood glucose level in order to pick-up the right time point in which both blood and skin are sensitives to increased glucose concentration. The next step is to find out glucose-responsive proteins or genes, then label them using the genome-editing technology. The third step consists of the integration of stem cells (iPSC) containing the biomarkers into the skin and then verifying whether the pre-determined biomarkers are responding to high blood glucose level. The last step is to develop an innovative device based on the confirmed biomarkers.

Supplementary Table I.

Primers sequences used for quantitative real-time PCR

Target gene	Primer Sequence (5` to 3`)	Taq Man Assay ID ™
**Glut-1**	**F: GCTGTGCTTATGGGCTTCTC**	
	**R: CACATACATGGGCACAAAGC**	
**MCU**	**F: GACCTCCTAAGCCATGAAGATG**	
	**R: AGCTCCCGCTCTTTGTTAAG**	
**MICU1**	**F: AATTGCCCAGGAACGAGAAA**	
	**R: GAGGACTGTTGTGAGGAAGATG**	
**Tubulin**	**F: GAGTGCATCTCCATCCACGTT**	
	**R: TAGAGCTCCCAGCAGGCATT**	
**Trisk 95**		**Mm00661493_m1**
**RYR-1**		**Mm01175211_m1**
**CASQ-2**		**Mm00486742_m1**
**18S**		**Mm04277571_s1**
**GAPDH**		**Mm99999915_g1**

Supplementary materials and methods

RNA extraction from skin samples and primary keratinocytes

Total RNA was extracted from the skin using Trizol (Invitrogen, 15596-026) and subsequently the RNeasy Mini Kit (Quiagen). The tissues were cut into small pieces, incubated with Trizol for 5 min at 4°C and homogenized using TissuLyser^{TM} (Qiagen) for 5 min at 50 Hz. Skin lysates were centrifuged for 15 min at 13000 rpm at 4°C, after which chloroform was added to the supernatant and the samples incubated for 5 min at RT followed by centrifugation for 18 min at 13000 rpm at 4°C. The rest of the extraction steps were performed using the RNeasy Mini Kit according to the manufacturer's instructions. The RNeasy Mini Kit was also used to purify the RNA from the primary keratinocytes. The quality of the RNA from both the skin and the cells was assessed using nanodrop. To analyse the mRNA expression level of *Trisk 95,* 2 µg of RNA was reverse transcribed to cDNA using the SuperScript™ VILO™ cDNA Synthesis Kit (ThermoFisher, 11754050) or the First Strand cDNA synthesis kit (Roche Applied Science). Quantitative real-time PCR was carried out for *Glut-1*, *Trisk 95*, *RYR-1*, *CSQ-2*, *MCU*, *MICU1*, *18S* and *GAPDH* using primers from a TaqMan gene expression assay and the SYBER Green method. The primer sequences used are listed in supplementary Table .I. The reactions were cycled 40 times after initial polymerase activation (50°C, 2 minutes) and initial denaturation (95°C, 20 minutes) using the following parameters: denaturation at 95°C for 1 second, and annealing and extension at 60°C for 20 seconds. The relative expression of target genes was normalized to *18S* or *GAPDH* expression and the ΔΔCT method was applied followed by a two-tailed Student's t-test.

Proteomic analysis

Sample preparation and protein digestion

Ten µg of each protein sample were solubilized in Laemlli buffer and deposited on SDS-PAGE gel for concentration and cleaning purposes. Separation was stopped once the proteins had entered the resolving gel. After colloidal blue staining, bands were cut out from the SDS-PAGE gel and subsequently cut into 1 mm x 1 mm pieces. These gel pieces were destained in 25 mM ammonium bicarbonate, 50% ACN, rinsed twice in ultrapure water and shrunk in ACN for 10 min. After ACN removal, the gel pieces were dried at room temperature, covered with trypsin solution (10 ng/µl in 40 mM NH4HCO3 and 10% ACN), rehydrated at 4°C for 10 min, and finally incubated overnight at 37°C. Spots were then incubated for 15 min in 40 mM NH4HCO3 and 10% ACN at room temperature with rotary shaking.

The supernatant was collected, and an H2O/ACN/HCOOH (47.5:47.5:5) extraction solution was added to the gel slices for 15 min. The extraction step was repeated twice, and the supernatants were pooled and concentrated in a vacuum centrifuge to a final volume of 100 µL. The digests were finally acidified by the addition of 2.4 µL of formic acid (5%, v/v) and stored at -20 °C.

nLC-MS/MS analysis

Peptide mixtures were analysed on an Ultimate 3000 nanoLC system (Dionex, Amsterdam, Netherlands) coupled to an Electrospray Q-Exactive Quadrupole Orbitrap benchtop mass spectrometer (Thermo Fisher Scientific, San Jose, CA). Ten microlitres of peptide digests were loaded onto a 300-µm inner diameter x 5-mm C_{18} PepMap^{TM} trap column (LC Packings) at a flow rate of 30 µL/min. The peptides were eluted from the trap column onto an analytical 75-mm id x 25-cm C18 Pep-Map column (LC Packings) with a 4–40% linear gradient of solvent B in 108 min (solvent A was 0.1% formic acid in 5% ACN, and solvent B was 0.1% formic acid in 80% ACN). The separation flow rate was set at 300 nL/min.

The mass spectrometer operated in positive ion mode at a 1.8-kV needle voltage. Data were acquired using Xcalibur 2.2 software in a data-dependent mode. MS scans (*m/z* 350-1600) were recorded at a resolution of R = 70 000 (@ m/z 200) and an AGC target of 3 x 10^6 ions collected within 100 ms. Dynamic exclusion was set to 30 s and the top 12 ions were selected after fragmentation in HCD mode. MS/MS scans with a target value of 1 x 10^5 ions were collected with a maximum fill time of 100 ms and a resolution of R = 17500. Only +2 and +3 charged ions were selected for fragmentation. The other settings were as follows: no sheath nor auxiliary gas flow, heated capillary temperature 250°C, normalized HCD collision energy 25% and isolation width 2 m/z.

Database search and processing of the results

Data were searched by SEQUEST through Proteome Discoverer 1.4 (Thermo Fisher Scientific Inc.) against a subset of the 2016.07 version of the UniProt database restricted to the Mus musculus Reference Proteome Set (49153 entries). Spectra from peptides higher than 5000 Da or lower than 350 Da were rejected. The search parameters were as follows: mass accuracies of the monoisotopic peptide precursor and peptide fragments were set to 10 ppm and 0.02 Da, respectively.

Only b- and y-ions were considered for the mass calculations. The oxidation of methionines (+16 Da) was considered to be a variable modification and the carbamidomethylation of cysteines (+57 Da) to be a fixed modification. Two missed trypsin cleavages were allowed. Peptide validation was

performed using the Percolator algorithm (Supplementary ref. [5]) and only “high confidence” peptides were retained, corresponding to a 1% False Positive Rate at the peptide level.

Label-Free Quantitative Data Analysis

Raw LC-MS/MS data were imported in Progenesis QI for Proteomics 2.0 (Nonlinear Dynamics Ltd, Newcastle, U.K). Data processing includes the following steps: (i) Features detection, (ii) Features alignment across 12 samples, (iii) Volume integration for 2-6 charge-state ions, (iv) Normalization on total protein abundance, (v) Import of sequence information, (vi) Calculation of protein abundance (sum of the volume of corresponding peptides), (vii) A t-test was calculated for each group comparison and proteins were filtered based on a p-value<0.05. Significantly, only non-conflicting features and unique peptides were considered for calculation at the protein level. Quantitative data were considered for proteins quantified in a minimum of 2 peptides.

ACN Acetonitrile
Da Dalton
CID Collision Induced Dissociation
SDS Sodium dodecyl sulphate

Supplementary References

1. Jensen, K. B., Driskell, R. R. & Watt, F. M. Assaying proliferation and differentiation capacity of stem cells using disaggregated adult mouse epidermis. *Nat. Protoc.* **5**, 898-911 (2010).
2. Marty, I. *et al*. Cloning and characterization of a new isoform of skeletal muscle triadin. *J. Biol. Chem.* **275**, 8206-8212 (2000).
3. Rezvani, H. R. *et al*. XPC silencing in normal human keratinocytes triggers metabolic alterations that drive the formation of squamous cell carcinomas. *J. Clin. Invest.* **121**, 195-211 (2011).
4. Prunskaite-Hyyrylainen, R. *et al*. Wnt4 coordinates directional cell migration and extension of the Mullerian duct essential for ontogenesis of the female reproductive tract. *Hum. Mol. Genet.* **25**, 1059-1073 (2016).
5. Kall, L., Canterbury, J. D., Weston, J., Noble, W. S. & MacCoss, M. J. Semi-supervised learning for peptide identification from shotgun proteomics datasets. *Nat. Methods* **4**, 923-925 (2007).
